# A Human Immuno‐Lung Organoid Model to Study Macrophage‐Mediated Lung Cell Senescence Upon SARS‐CoV‐2 Infection

**DOI:** 10.1002/advs.202503932

**Published:** 2025-07-25

**Authors:** Yuling Han, Dongliang Leng, Tuo Zhang, Jian Ge, Yinshan Fang, Tiankun Lu, Xue Dong, Manoj S Nair, Neranjan de Silva, Zhaowei Han, Tiancheng Jiao, Yuanhao Huang, Meiqi Zhao, Anjali Saqi, Hanina Hibshoosh, Zihe Meng, Jenny Z Xiang, Chendong Pan, Yanjie Sun, David D. Ho, Todd Evans, Jie Liu, Liuliu Yang, Jianwen Que, Shuibing Chen

**Affiliations:** ^1^ Department of Surgery Weill Cornell Medicine 1300 York Ave New York NY 10065 USA; ^2^ Center for Genomic Health Weill Cornell Medicine 1300 York Ave New York NY 10065 USA; ^3^ Genomic Resource Core Facility Weill Cornell Medicine New York NY 10065 USA; ^4^ Columbia Center for Human Development and Division of Digestive and Liver Disease Department of Medicine, Vagelos College of Physicians and Surgeons Columbia University IrvingMedical Center New York NY 10032 USA; ^5^ Aaron Diamond AIDS Research Center Columbia University Vagelos College of Physicians and Surgeons New York NY 10032 USA; ^6^ Gilbert S. Omenn Department of Computational Medicine & Bioinformatics University of Michigan Ann Arbor MI 48109 USA; ^7^ Department of Pathology and Cell Biology Columbia University Irving Medical Center New York NY 10032 USA

**Keywords:** macrophage, organoid, spatial transcriptomics

## Abstract

While COVID‐19 affects multiple organ systems, the human respiratory system is the primary viral target and main site for disease progression. In this study, spatial transcriptional assays (NanoString CosMx) are utilized to analyze both explant and autopsy samples from non‐COVID and COVID‐19 lungs, identifying the activation of proinflammatory macrophages in COVID‐19 explants. It is further developed immuno‐lung organoids comprising hPSC‐derived alveolar and airway organoids co‐cultured with macrophages to investigate the impact and underlying mechanisms of macrophage‐mediated lung damage following SARS‐CoV‐2 infection. The findings demonstrate that proinflammatory macrophages induce lung cell senescence through the THBS1–(ITGA3+ITGB1) signaling axis, a mechanism further validated using spatial transcriptomics. This study not only establishes physiologically relevant immuno‐lung organoid models for modeling macrophage‐mediated tissue damage, but also identifies a previous unrecognized role of the THBS1‐(ITGA3+ITGB1) pathway in driving lung cell senescence during infectious disease.

## Graphical abstract

Immuno‐lung organoids are developed and used spatial transcriptomics to uncover how proinflammatory macrophages drive lung cell senescence via the THBS1–(ITGA3+ITGB1) axis in COVID‐19. To ensure broad accessibility, LungSpatialDB is created, an AI‐powered data portal, enabling intuitive exploration of spatial data and biological insights through natural language queries.

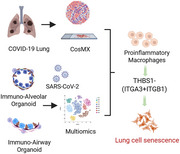

## 1 Introduction

SARS‐CoV‐2 primarily targets the respiratory tract. The infection of alveolar epithelial type 2 (AT2) cells initiates a cascade of lung injury characteristic of COVID‐19 pneumonia. The virus induces diffuse lung damage, and in severe cases, the disease progresses to acute respiratory distress syndrome (ARDS). These pathological changes result from a combination of direct viral cytopathic effects and a maladaptive immune response, marked by immune cell infiltration, cytokine storms, and disruption of the endothelial barrier. This multi‐step process ultimately culminates in respiratory failure, the leading cause of mortality in COVID‐19 patients. Elucidating the mechanisms underlying immune cell–mediated lung injury is critical for understanding disease progression and for identifying potential therapeutic targets.^[^ ^1^ ^]^

Spatial technologies, including spatial transcriptomics, and imaging mass cytometry (IMC), have been employed to analyze lung autopsy samples from COVID‐19 patients. For example, IMC analysis revealed neutrophil and macrophage extravasation in COVID‐19 lung autopsy samples.^[^ ^2^ ^]^ In addition, NanoString GeoMx analysis comparing lung autopsy samples of fatal influenza and COVID‐19 patients identified a limited number of differentially expressed genes, including interferon‐associated gene *IFI27*, a known blood biomarker distinguishing bacterial and viral lung infections.^[^ ^3^ ^]^ Another in situ hybridization (ISH) tissue analysis of lung autopsies showed that cytotoxic lymphocytes expressing IFNG, which induces chemokines to promote the recruitment of macrophages.^[^ ^4^ ^]^ SARS‐CoV‐2 infection damages host cells through both direct virus‐induced cytopathic effect and by triggering immune responses. Human pluripotent stem cells (hPSCs) offer a versatile in vitro platform for dissecting infectious disease mechanisms.^[^ ^5^ ^]^

Using hPSC‐derived cells and organoids, we have established models to study viral tropism.^[^ ^6^ ^]^ and developed SARS‐CoV‐2 infection models in alveolar^[^ ^7^ ^]^ and airway organoids,^[^ ^8^ ^]^ which have been applied to drug screening and analysis of virus–host interactions. Macrophage infiltration has been reported in the lungs,^[^ ^9^ ^]^ heart,^[^ ^10^ ^]^ and pancreas^[^ ^11^ ^]^ of COVID‐19 patients. To explore immune‐mediated cardiac injury, we previously established a 2D co‐culture system of hPSC‐derived cardiomyocytes and macrophages^[^ ^10^ ^]^ and a vascularized macrophage–islet organoid model to demonstrate that proinflammatory macrophages drive β‐cell pyroptosis.^[^ ^11^ ^]^ However, an immuno‐lung organoid model for studying infectious diseases has been lacking. In this study, we developed immuno‐lung organoids composed of hPSC‐derived macrophages and alveolar/airway organoids to investigate macrophage‐mediated lung cell damage.

We first applied spatial transcriptomics (NanoString CosMx) to analyze explant and autopsy lung samples from non‐COVID and COVID‐19 patients, revealing prominent activation of proinflammatory macrophages in COVID‐19 lung explants. To investigate the impact of proinflammatory macrophages on lung cells and elucidate the underlying mechanism, we developed immuno‐lung organoids composed of either unspecific or proinflammatory macrophages. Our findings demonstrate that proinflammatory macrophages induce lung cell senescence through the THBS1–(ITGA3+ITGB1) signaling axis, a mechanism further validated by spatial transcriptomic analysis.

## 2 Results

### 2.1 Spatial Transcriptome Analysis to Identify the Accumulation of Macrophages in Distal Lung from COVID‐19 Patients

**Figure 1:**
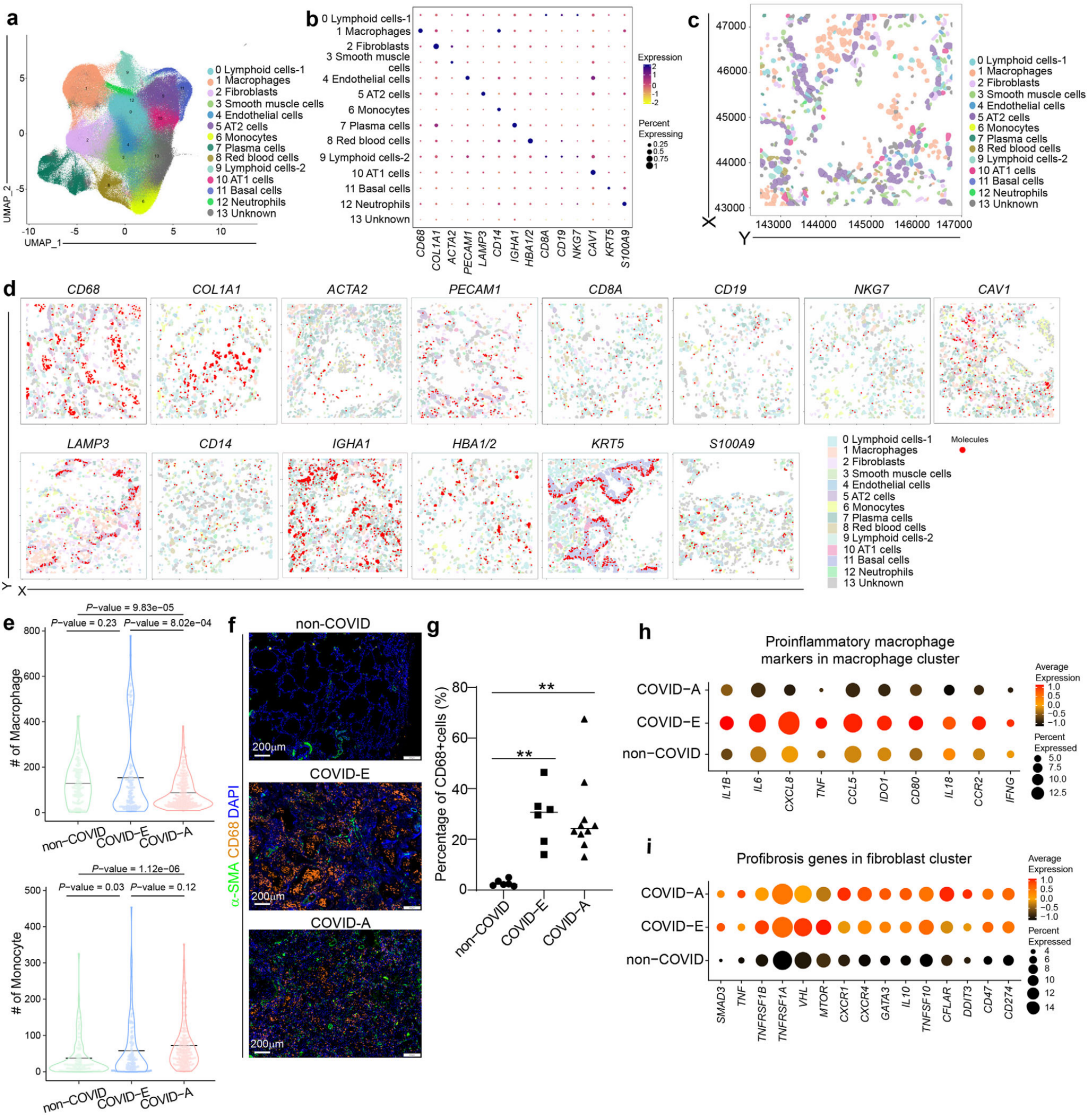
Spatial transcriptomics analysis of lung explant and autopsy samples from COVID‐19 patients. a) UMAP of non‐COVID (N=6), COVID‐E (N=6), COVID‐A (N=10) lung samples. b) Dot plot of marker gene expression in each cluster of non‐COVID (N=6), COVID‐E (N=6), COVID‐A (N=10) lung samples. c) Representative image plot shows the cell types in a non‐COVID sample. d) Representative image plots show the marker gene expression of each cluster of non‐COVID (N=6), COVID‐E (N=6), COVID‐A (N=10) lung samples. e) Quantification of macrophages and monocytes in non‐COVID (N=6), COVID‐E (N=6), COVID‐A (N=10) lung samples. f) Representative images of immunostaining of CD68 and a‐SMA in non‐COVID, COVID‐E, COVID‐A lung samples. Scale bar=20 µm. g) Quantification of CD68+ macrophages in in non‐COVID (N=6), COVID‐E (N=6), COVID‐A (N=10) lung samples. h) Dot plot analysis of proinflammatory macrophage‐associated genes in the macrophage cluster of non‐COVID (N=6), COVID‐E (N=6), COVID‐A (N=10) lung samples. i) Dot plot analysis of pulmonary fibrosis‐associated genes in the fibroblast cluster of non‐COVID (N=6), COVID‐E (N=6), COVID‐A (N=10) lung samples.

To systematically analyze lung damage and immune cell infiltration in COVID‐19 patients, we collected human lung explant tissues from COVID‐19 patients who underwent lung transplantation (COVID‐E), as well as lung autopsy tissues from COVID‐19 patients (COVID‐A) and age and gender matched non‐COVID‐19 controls (non‐COVID) (**Extended Data** Figure S1a; Table S1, Supporting Information). Compared to non‐COVID controls, COVID‐E and COVID‐A samples showed significant pathological changes, including disrupted lung structure and mononuclear cell infiltration (**Extended Data** Figure S1b, Supporting Information). To further investigate the spatial transcriptional changes, CosMx, a spatial single‐cell transcriptional profiling platform was applied to analyze the lung tissue sections from 22 donors. We selected 12‐24 field of views (FOVs) per sample, totaling 400 FOVs crossing 22 samples. Using a custom‐designed pipeline (**see Experimental Section**), we identified 420,426 cells. UMAP projection identified a variety of cell types, including lymphocytes (*CD8A*+, *CD19*+, or *NKG7*+), macrophages (*CD68*+), fibroblasts (*COL1A1*+), smooth muscle cells (*ACTA2*+), endothelial cells (*PECAM1*+), AT2 cells (*LAMP3*+),^[^ ^12^ ^]^ monocytes (*CD14*+), plasma cells (*IGHA1*+), red blood cells (*HBA1/2*+), AT1 cells (*CAV1*+),^[^ ^13^ ^]^ basal cells (*KRT5*+), and neutrophils (*S100A9*+) (**Figure** **1**a–d; **Extended Data** Figure S1c, Supporting Information). Lymphocytes were classified into two subtypes due to the spatial separation of the two clusters on the UMAP (Figure 1a).

Given the extensive work done to study the impact of direct SARS‐CoV‐2 infection on lung organoids and tissues,^[^ ^7^, ^14^, ^15^ ^]^ we focused instead on the immune cell components to investigate immune‐mediated lung damage. We analyzed the presence of macrophages, monocytes, lymphocytes, and neutrophils in non‐COVID, COVID‐E and COVID‐A samples. We found that the enrichment of macrophages and monocytes (Figure 1e), but not lymphocytes or neutrophils in COVID‐E samples compared to non‐COVID samples (**Extended Data** Figure S1d,e, Supporting Information). Immunostaining further confirmed the enrichment of macrophages in COVID‐E samples (Figure 1f,g; **Extended Data** Figure S1f, Supporting Information). Next, we focused on the macrophage cluster and observed increased expression levels of proinflammatory macrophage related genes, including *IL1B, IL6, CXCL8, TNF, CCL5, IDO1, CD80, CCR2, IFNG* (Figure 1h), in COVID‐E, but not COVID‐A samples. For comparison, we also monitored the expression of fibrosis associated genes, previously reported in COVID‐19 studies,^[^ ^16^ ^]^ observed upregulation of these genes in both COVID‐E and COVID‐A samples (Figure 1i). Together, these results suggest that the upregulation of proinflammatory macrophages is only detected in COVID‐E samples, but not COVID‐A samples, emphasizing the invaluable information obtained through studying fresh explant tissues.

To ensure this comprehensive dataset is publicly accessible, we have developed an AI‐powered user interface, LungSpatialDB (available at www.lungspatialdb.com), which leverages the advanced capabilities of the Large Language Model ChatGPT‐4o. This AI assistant, designed through sophisticated prompt engineering, enables users to interact with the platform through natural language queries. Its key features include providing detailed background information on biological terms, presenting experimental results from LungSpatialDB, demonstrating platform capabilities through illustrative examples, and offering comprehensive introductions to the website. By addressing diverse user needs with remarkable adaptability, the AI assistant enhances the user experience and ensures the seamless accessibility of LungSpatialDB’s rich dataset.

### 2.2 Construction of an Immuno‐Lung Organoid Model

To investigate the role of macrophages on lung epithelial cell damage of COVID‐19 patients, we developed hPSC‐derived macrophage‐alveolar organoid (AVM organoid) models, using hPSC‐derived alveolar organoids and hPSC‐derived macrophages, respectively (**Extended Data** Figure S2a, Supporting Information). hPSCs were first differentiated into alveolar epithelial cells and cultured as organoids following previously published protocols.^[^ ^7^ ^]^ Next, macrophages were derived from RFP+ hPSCs using our published protocol.^[^ ^6^ ^]^ After optimizing the culture medium and cell ratio, we combined the alveolar organoid clusters with macrophages in a 3D culture to form organoids (**Extended Data** Figure S2b, Supporting Information). RFP+ macrophages can be detected in the AVM organoids and maintained for over two weeks in vitro (**Extended Data** Figure S2b, Supporting Information). Electron microscopy analysis confirmed the presence of lamellar bodies within AT2 cells in the alveolar organoids (**Extended Data** Figure S2c, Supporting Information).

**Figure 2:**
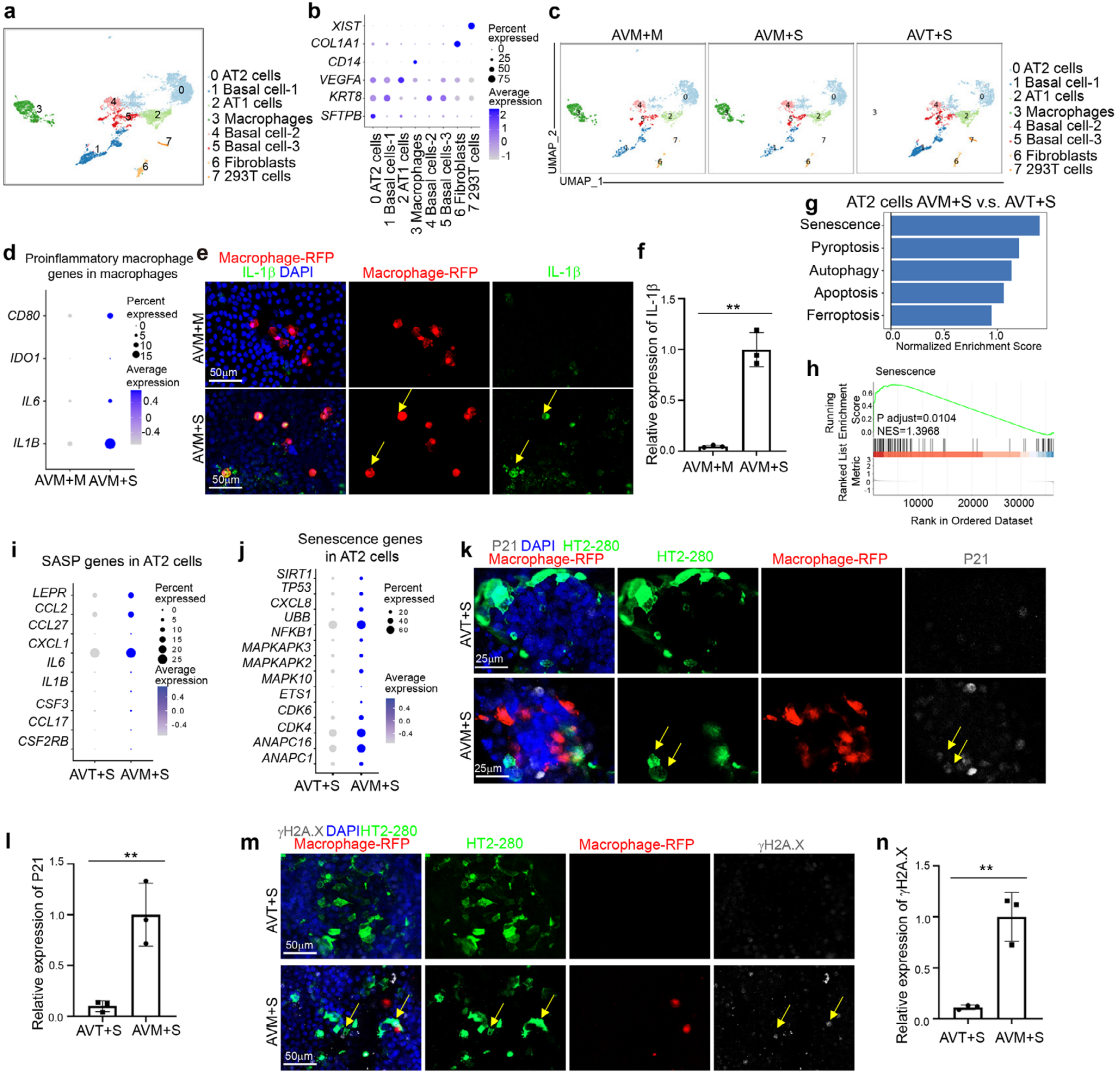
hPSC‐derived immuno‐alveolar organoids to study macrophage‐mediated lung damage during SARS‐CoV‐2 infection. a) UMAP of hPSC‐derived immuno‐alveolar organoids analyzed by scRNA‐seq. b) Dot plot displaying cell marker gene of each cluster of hPSC‐derived immuno‐alveolar organoids. c) Individual UMAP of immuno‐alveolar organoids exposed to mock (AVM+M) or SARS‐CoV‐2 (MOI=0.25, AVM+S), and alveolar organoids co‐cultured with 293T cells exposed to SARS‐CoV‐2 (MOI=0.25, AVT+S). d) Dot plot analysis of proinflammatory macrophage‐associated genes in macrophage cluster of AVM+M and AVM+S conditions. e,f) Immunostaining (e) and quantification (f) of the relative expression of IL‐1β in RFP^+^ macrophages of AVM+M and AVM+S conditions. The yellow arrows highlight the expression of IL1‐B in RFP^+^ macrophages. Scale bar= 50 µm. g) Enrichment of cell death pathways in AT2 cell cluster of immuno‐alveolar organoids (AVM+S) or 293T co‐cultured with alveolar organoids (AVT+S) exposed to SARS‐CoV‐2 (MOI=0.25). h) Gene Set Enrichment Analysis (GSEA) of senescence pathway in AT2 cell cluster of AVM+S versus AVT+S condition. i) Dot plot analysis of senescence‐associated secretory phenotype (SASP) associated genes in AT2 cell cluster of AVM+S and AVT+S conditions. j) Dot plot analysis of senescence associated genes in AT2 cell cluster of AVM+S and AVT+S conditions. k,l) Immunostaining (k) and quantification (l) of the relative expression of p21 in of AVM+S and AVT+S conditions. The yellow arrows highlight the expression of p21 in HT2‐280^+^ AT2 cells. Scale bar= 50 µm. m,n) Immunostaining (m) and quantification (n) of the relative expression of γH2A.X in of AVM+S and AVT+S conditions. The yellow arrows highlight the expression of γH2A.X in HT2‐280^+^ AT2 cells. Scale bar= 50 µm. N=3 independent biological replicates. Data was presented as mean ± STDEV. *P* values were calculated by unpaired two‐tailed Student's *t*‐test. **P < 0.01.

To further examine the status of macrophages upon virus exposure, we performed single cell RNA‐seq (scRNA‐seq) of AVM organoids that were exposed to SARS‐CoV‐2 (AVM+S). Two controls were included, AVM upon mock infection (AVM+M), and alveolar organoid that were co‐cultured with 293T cells and exposed to SARS‐CoV‐2 infection (AVT+S) (**Extended Data** Figure S2a, Supporting Information). 293T cells were used as a control to normalize multiplicity of infection (MOI). UMAP and dot plot analysis identified eight cell clusters, including *SFTPB*+ AT2 cells, *KRT8*+ basal cells, *VEGFA*+ AT1 cells,^[^ ^17^ ^]^ *CD163*+ macrophages, *COL1A1*+ fibroblasts and *XIST*+ 293T cells (**Figure** **2**a–c; **Extended Data** Figure S2d–f, Supporting Information). The viral reads in AVM+S and AVT+S condition confirmed the robust viral infection (**Extended Data** Figure S3a, Supporting Information). We first analyzed the macrophage cluster and found increased expression levels of proinflammatory macrophage‐associated genes, including *CD80, IL1B*, *IDO1* and *IL6*, in the SARS‐CoV‐2 exposed group (AVM+S) compared to mock condition (AVM+M) (Figure 2d). Immunostaining further confirmed the upregulation of the proinflammatory macrophage marker, IL1β, in the macrophage cluster for the AVM+S condition (Figure 2e,f). Next, we analyzed the AT2 cell cluster and performed Gene Set Enrichment Analysis (GSEA) of pathways associated with cell death and cell stress. We found that the senescence pathway was activated in AT2 cells within AVM organoids infected with SARS‐CoV‐2 (AVM+S), but not in the non‐macrophage control (AVT+S) or in AVM organoids without SARS‐CoV‐2 infection (AVM+M) (Figure 2g,h). Dot plots further demonstrated that senescence‐associated secretory phenotype (SASP) genes and other senescence pathway‐related genes were upregulated in AT2 cells from AVM+S organoids compared to both AVT+S (Figure 2i,j) and AVM+M conditions (**Extended Data** Figure S3b,c, Supporting Information). Immunostaining also confirmed the upregulation of P21 and γH2A.X in HT2‐280^+^ AT2 cells for the AVM+S condition compared to AVT+S (Figure 2k–n; **Extended Data** Figure S3d,e, Supporting Information) and AVM+M conditions (**Extended Data** Figure S3f–i, Supporting Information). In addition, we also detected similar P21 and γH2A.X upregulation in non AT2 (HT2‐280‐) cells, suggesting that cell senescence is not specific to AT2 cells. Consistently, the senescence pathway was activated in the basal cell cluster in AVM organoids infected with SARS‐CoV‐2 (AVM+S) but not non‐macrophage control (AVT+S) (**Extended Data** Figure S3j, Supporting Information). Consistent with the spatial transcriptomics data, pulmonary fibrosis‐associated genes were also upregulated in the fibroblast cluster for AVM+S compared to AVT+S conditions (**Extended Data** Figure S3k, Supporting Information).

In addition to immuno‐alveolar organoids, we also constructed the immuno‐airway organoids containing hPSC‐derived airway organoids and hPSC‐derived macrophages. Like immuno‐alveolar organoids, the immuno‐airway organoids were exposed to SARS‐CoV‐2 (ARM+S) and analyzed with scRNA‐seq. The immuno‐airway organoids with mock infection (ARM+M) and airway organoids co‐cultured with 293T cells upon SARS‐CoV‐2 exposure (ART+S) were used as controls (**Extended Data** Figure S4a, Supporting Information). UMAP analysis revealed seven cell clusters, including *KRT5*+ basal cells, *TUBA1A*+ ciliated cells, *CD163*+ macrophages, *KRT18*+ proliferating basal cells, *COL1A1*+ fibroblasts and *XIST*+ 293T cells (**Extended Data** Figure S4b–e, Supporting Information). We compared the transcriptional profiles of macrophages and observed increased expression levels of proinflammatory macrophage associated genes, including *CD80*, *IL1B* and *IL6* in the macrophage cluster for the ARM+S condition (**Extended Data** Figure S5a,b, Supporting Information). Immunostaining further confirmed the upregulation of proinflammatory macrophage marker, IL1β, in the macrophage cluster for the ARM+S condition (**Extended Data** Figure S5c,d, Supporting Information). In addition, dot plots analysis revealed that SASP and senescence pathway associated genes were upregulated in ciliated cells of ARM+S sample compared to ART+S sample (**Extended Data** Figure S5e,f, Supporting Information). Moreover, immunostaining confirmed the upregulation of P21 and γH2A.X in ciliated cells for the ARM+S condition (**Extended Data** Figure S5g–n, Supporting Information).

### 2.3 Proinflammatory Macrophages Induce Lung Cell Senescence

**Figure 3:**
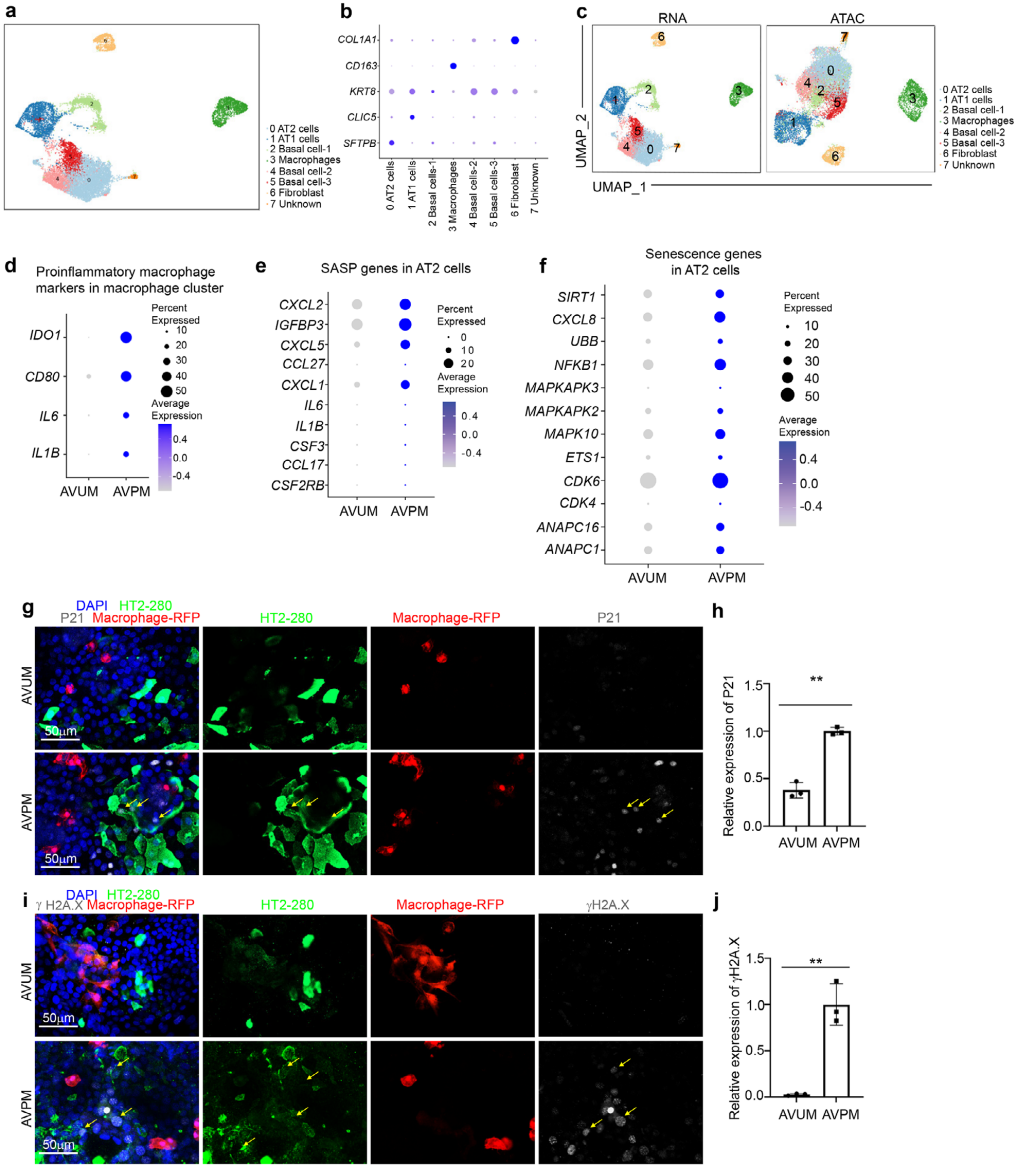
Construction and sn‐multiomics analysis of alveolar organoids containing unstimulated or proinflammatory macrophages. a) UMAP of hPSC‐derived immuno‐alveolar organoids containing unstimulated (AVUM) or proinflammatory macrophages (AVPM). b) Dot plot displaying cell marker genes. c) Individual UMAP of snRNA‐seq and snATAC‐seq analysis of hPSC‐derived AVUM or AVPM organoids. d) Dot plot analysis of proinflammatory macrophage‐associated genes in macrophage cluster of hPSC‐derived AVUM or AVPM organoids. e) Dot plot analysis of SASP associate genes in AT2 cell cluster of hPSC‐derived AVUM or AVPM organoids. f) Dot plot analysis of senescence associate genes in AT2 cell cluster of hPSC‐derived AVUM or AVPM organoids. g,h) Immunostaining (g) and quantification (h) of the relative expression of p21 in of hPSC‐derived AVUM or AVPM organoids. The yellow arrows highlight the expression of p21 in HT2‐280^+^ AT2 cells. Scale bar= 50 µm. i,j) Immunostaining (i) and quantification (j) of the relative expression of γH2A.X in hPSC‐derived AVUM or AVPM organoids. The yellow arrows highlight the expression of γH2A.X in HT2‐280^+^ AT2 cells. Scale bar= 50 µm. N=3 independent biological replicates. Data was presented as mean ± STDEV. *P* values were calculated by unpaired two‐tailed Student's t test. **P < 0.01.

We demonstrated the activation of proinflammatory macrophages and the upregulation of the senescence pathway in immuno‐lung organoids when exposed to SARS‐CoV‐2. To determine whether proinflammatory macrophages induce cell senescence in respiratory cells, we constructed the immuno‐alveolar organoids using either proinflammatory (AVPM) or unstimulated macrophages (AVUM), both of which were analyzed using single‐nuclear multiomics (sn‐multiomics) (**Extended Data** Figure S6a, Supporting Information). UMAP identified *SFTPB*+ AT2 cells, *CLIC5*+ AT1 cells, *KRT8*+ basal cells, *CD163*+ macrophages, *COL1A1*+ fibroblasts (**Figure** **3**a–c; **Extended Data** Figure S6b, Supporting Information). Dot plot showed the increased expression levels of *IDO1, CD80, IL6* and *IL1B* in the macrophage cluster of AVPM organoids, which confirms the identities of proinflammatory macrophages (Figure 3d). Moreover, dot plot analysis revealed the upregulation of SASP and senescence pathway associated genes in the AT2 cell cluster of AVPM organoids (Figure 3e,f). Furthermore, immunostaining validated the upregulation of P21 and γH2A.X in AT2 cells of AVPM organoids (Figure 3g–j). Similar to the AVM+S condition, senescence markers were also detected in HT2‐280‐ cells, suggesting that proinflammatory macrophage‐mediated senescence is not limited to AT2 cells.

We next constructed immuno‐airway organoids containing either proinflammatory (ARPM) or unstimulated (ARUM) macrophages to confirm that proinflammatory macrophages also induce lung airway organoid senescence (**Extended Data** Figure S7a, Supporting Information). sn‐multiomics analysis of ARPM and ARUM organoids identified five cell clusters, including *KRT17*+ basal cells, *TUBA1A*+ ciliated cells, *CD163*+ macrophages and *COL1A1*+ fibroblasts (**Extended Data** Figure S7b–e, Supporting Information). The proinflammatory macrophages maintained their cellular identities, as confirmed by the expression of the proinflammatory macrophage marker genes, including *CD80*, *IL6*, and *IL1B* (**Extended Data** Figure S7f, Supporting Information). Dot plot analysis also revealed upregulation of the SASP and senescence pathway associated genes in the ciliated cells present in ARPM organoids (**Extended Data** Figure S7g,h, Supporting Information). The upregulation of senescence phenotypes was further confirmed by staining with P21 and gH2A.X antibodies (**Extended Data** Figure S7i–l, Supporting Information).

### 2.4 THBS1‐(ITGA3+ITGB1) Pathway Contributes to Proinflammatory Macrophage‐Mediated Lung Cell Senescence

**Figure 4:**
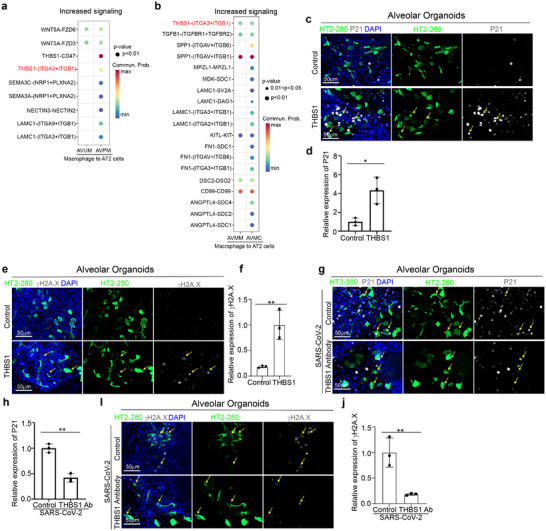
Proinflammatory macrophages cause lung cell senescence by the THBS1‐(ITGA3+ITGB1) pathway. a) Dot plot shows the upregulated signals from macrophages to AT2 cells in alveolar organoids containing unstimulated (AVUM) or proinflammatory macrophages (AVPM). b) Dot plot shows the upregulated signals from macrophages to AT2 cells in immuno‐alveolar organoids exposed to mock (AVM+M) versus SARS‐CoV‐2 (MOI=0.25, AVM+S). c,d) Immunostaining (c) and quantification (d) of the relative expression of p21 in hPSC‐derived alveolar organoids treated with control or 5 µg mL^−1^ THBS1 protein. The yellow arrows highlight the expression of p21 in HT2‐280^+^ AT2 cells. Scale bar= 50 µm. e,f) Immunostaining (e) and quantification (f) of the relative expression of γH2A.X in hPSC‐derived airway organoids treated with control or 5 µg mL^−1^ THBS1 protein. The yellow arrows highlight the expression of p21 in HT2‐280^+^ AT2 cells. Scale bar= 50 µm. g,h) Immunostaining (g) and quantification (h) of the relative expression of p21 in hPSC‐derived immuno‐alveolar organoids treated with control or 10 µg mL^−1^ THBS1 blocking antibody upon SARS‐CoV‐2 infection. The yellow arrows highlight the expression of p21 in HT2‐280^+^ AT2 cells. Scale bar= 50 µm. i,j) Immunostaining (i) and quantification (j) of the relative expression of γH2A.X in hPSC‐derived immuno‐alveolar organoids treated with control or 10 µg mL^−1^ THBS1 blocking antibody upon SARS‐CoV‐2 infection. The yellow arrows highlight the expression of γH2A.X in HT2‐280^+^ AT2 cells. Scale bar= 50 µm. N=3 independent biological replicates. Data was presented as mean ± STDEV. *P* values were calculated by unpaired two‐tailed Student's t test. *P < 0.05, **P < 0.01.

To determine the mechanisms by which proinflammatory macrophages induce lung cell senescence, we performed cell‐cell interaction (cell‐chat) analysis and focused on the signals transmitted from macrophages to AT2 cells in AVPM organoids. We first identified nine ligand‐receptor pairs that are enriched from macrophage to AT2 cells in AVPM organoids compared to AVUM organoids (**Figure** **4**a). Out of nine pairs, THBS1‐(ITGA3+ITGB1) is the only one that is enriched from macrophage to AT2 cell cluster of AVM organoids upon SARS‐CoV‐2 stimulation (AVM+S, Figure 4b). Consistently, THBS1‐(ITGA3+ITGB1) is also increased in the interaction from macrophages to ciliated cells in ARPM organoids (**Extended Data** Figure S8a, Supporting Information), as well as for the ARM+S condition (**F Extended Data** Figure S8b, Supporting Information). Next, we treated alveolar or airway organoids with THBS1 and confirmed that THBS1 induced cell senescence in alveolar or airway organoids as indicated by P21 and γH2A.X staining (Figure 4c–f; **Extended Data** Figure S8c–f, Supporting Information). In addition, blocking THBS1 with antibody significantly decreased macrophage‐mediated cell senescence in AVM+S and ARM+S conditions (Figure 4g–j; **Extended Data** Figure S8g–j, Supporting Information). These data indicate that the THBS1‐(ITGA3+ITGB1) pathway is a key mechanism contributing to proinflammatory macrophage mediated lung cell senescence upon viral infection.

### 2.5 Increased Lung Cell Senescence in COVID‐E Samples

**Figure 5:**
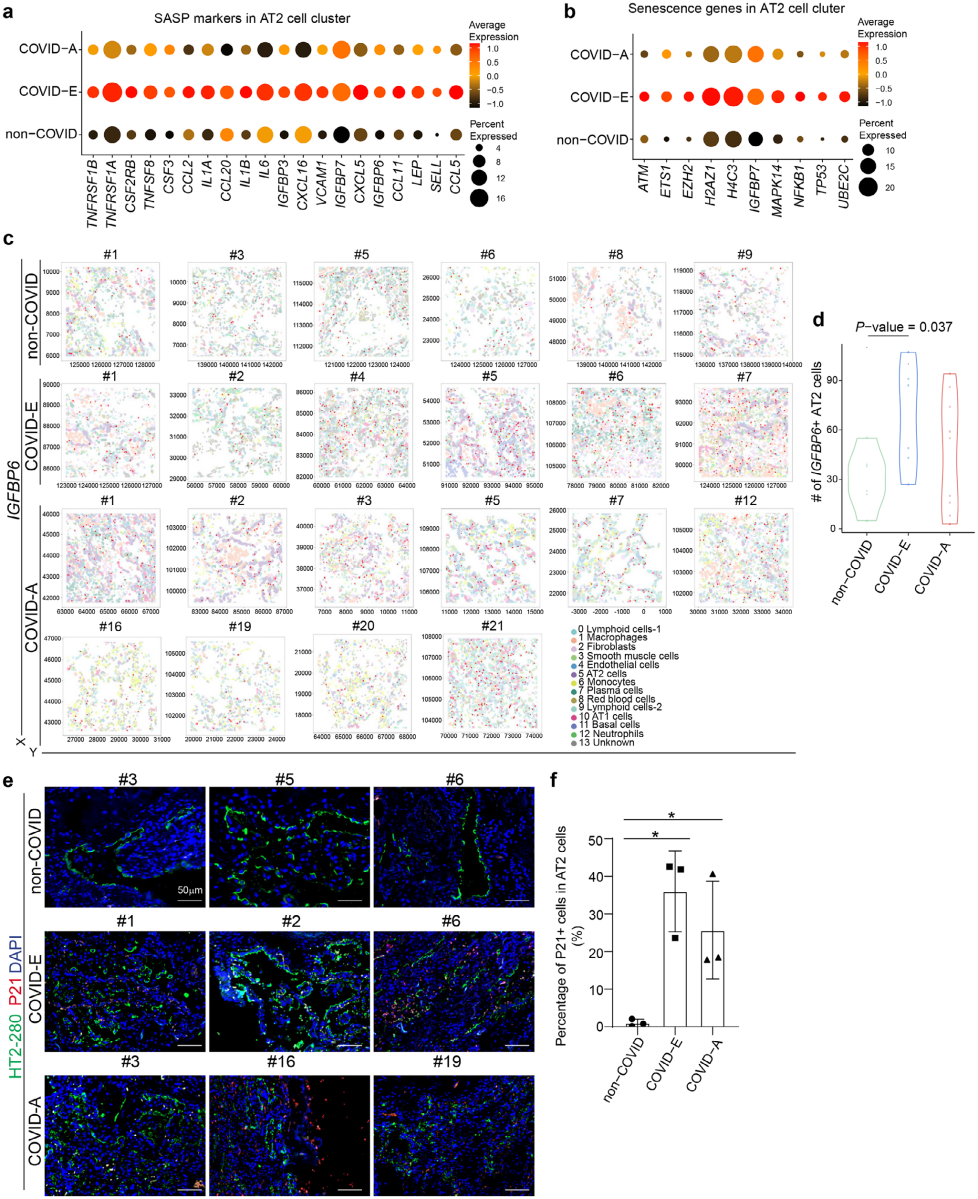
Senescence increases in lung cells of COVID‐19 explant samples. a) Dot plot analysis of SASP associate genes in AT2 cell cluster of non‐COVID (N=6), COVID‐E (N=6), COVID‐A (N=10) lung samples. b) Dot plot analysis of senescence associate genes in AT2 cell cluster of non‐COVID (N=6), COVID‐E (N=6), COVID‐A (N=10) lung samples. c,d) Representative image plots (c) and quantification (d) shows the expression of IGFBP6 in non‐COVID (N=6), COVID‐E (N=6), COVID‐A (N=10) lung samples. e,f) Immunostaining (e) and quantification (f) of the relative expression of p21 in non‐COVID (N=6), COVID‐E (N=6), COVID‐A (N=10) lung samples. The yellow arrows highlight the expression of p21 in HT2‐280^+^ AT2 cells. Scale bar= 50 µm. N=3 independent biological replicates. Data was presented as mean ± STDEV. *P* values were calculated by unpaired two‐tailed Student's t test. *P < 0.05.

We then validated the senescence phenotype in lung cells using non‐COVID, COVID‐E, and COVID‐A samples. Spatial transcriptomics analysis showed that the SASP associated genes and senescence pathway associated genes were upregulated in AT2 cells of COVID samples, especially the COVID‐E samples. (**Figure** **5**a,b). Image plots and violin plot also showed the increased expression of the senescence marker gene *IGFBP6* ^[^ ^18^, ^19^ ^]^ in human lung cells of explant samples when compared to control autopsy samples (Figure 5c,d). Consistent with our spatial transcriptome analysis, we also observed the extensive presence of P21+ cells in COVID‐E lung samples (Figure 5e,f).

## 3 Discussion

Spatial transcriptomics provides a powerful tool for monitoring disease progression. The technology helps us to elucidate the spatial heterogeneity of viral infection and immune responses, offering critical insights into disease mechanisms and aiding the development of effective therapeutic strategies. Several spatial transcriptomic analyses have been applied to study COVID‐19 autopsy samples, focused on lung,^[^ ^2^, ^4^, ^20^ ^]^ liver,^[^ ^21^ ^]^ heart,^[^ ^22^ ^]^ pancreas^[^ ^11^ ^]^ and placenta.^[^ ^23^ ^]^ Through analysis of patient lungs with high and low viral loads, a model of COVID‐19 pneumonia progression has been proposed. The early stage is characterized by high viral loads and infiltration of proinflammatory macrophages with a strong type I interferon (IFN) response, while the later stage involves tissue repair, viral clearance, and a diminished IFN response.^[^ ^24^ ^]^ In this study, we applied CosMx spatial transcriptomics to comprehensively profile immune cell dynamics and epithelial cell injury in COVID‐19 lung tissues. Importantly, we performed a parallel analysis of explant samples from patients undergoing lung transplantation (COVID‐E) and autopsy samples from deceased patients (COVID‐A), representing, to our knowledge, the first direct comparison of these two clinically distinct cohorts. Our analysis revealed a pronounced accumulation of proinflammatory macrophages and marked cellular senescence in COVID‐E samples, features that were notably less prominent in COVID‐A tissues (Figure 5). These differences likely reflect variations in disease stage and host immune response. Although patients in the COVID‐E group may have experienced disease severity comparable to those in the COVID‐A group, they likely benefited from timely and advanced clinical interventions. This observation underscores the importance of analyzing tissue specimens collected at distinct stages of disease progression to uncover dynamic mechanisms of SARS‐CoV‐2–induced lung injury. Previous studies reported the abundance of also CD163+ anti‐inflammatory/pro‐fibrotic macrophages in late‐stage COVID‐19.^[^ ^25^ ^]^ While we observed a robust accumulation of pro‐inflammatory macrophages in COVID‐E samples, discrepancies across studies may result from several factors, including differences in tissue procurement protocols, donor heterogeneity (e.g., disease severity and treatment history), and variations in analytical pipelines. These factors may contribute to the relative underrepresentation of CD163⁺ macrophages in our dataset.

Immune‐mediated host damage has been implicated in various diseases, including infections. There is an urgent need to develop suitable in vitro human models to study immune‐mediated host damage. Several immuno‐epithelial organoids have been developed using adult tissues. For example, human intestinal immuno‐organoids have been derived from human intestinal specimens.^[^ ^26^, ^27^ ^]^ Additionally, macrophages have been incorporated into a murine bronchioalveolar lung organoid derived from bronchioalveolar stem cells, to create murine macrophage‐lung organoid models.^[^ ^28^ ^]^ Moreover, mouse colorectal cancer organoids, derived from mice carrying KrasG12D/+ and/or Trp53R172H/– mutations, were cocultured with macrophages to study the impact of macrophages on tumorigenesis.^[^ ^29^ ^]^ However, immuno‐epithelial organoids derived from adult tissues are limited by the availability of starting materials, making it challenging to scale up production. An alternative approach to generating immuno‐epithelial organoids involves co‐development. For example, macrophages have been detected in hPSC‐derived intestinal organoids^[^ ^30^, ^31^ ^]^ or lung organoid^[^ ^29^, ^32^ ^]^ by co‐development. The co‐development model provides a robust platform for studying cell‐cell interactions during embryonic development. However, it remains challenging to control the ratio of immune cells to host tissue cells, as well as the specific identities of the immune cells, which limits its application in disease modeling. To address these limitations, we developed immuno‐lung organoid models incorporating hPSC‐derived alveolar/airway organoids and macrophages. This model allows for precise control of the ratio and cellular states of lung cells and macrophages. Moreover, it can be adapted for scalable production, enabling the study of molecular mechanisms underlying macrophage‐mediated host damage and facilitating drug screening.

scRNA‐seq analysis identified increased senescence of lung cells, including AT2 cells, within immuno‐alveolar organoids upon SARS‐CoV‐2 infection. The increased lung cell senescence was further confirmed in alveolar organoids co‐cultured with proinflammatory macrophages. CosMx analysis and immunostaining validated the upregulation of senescence in lung cells within lung explant samples. Consistent with our results, previous studies have shown that COVID‐19 patients exhibit markers of senescence in their airway mucosa in situ and have elevated serum levels of SASP factors.^[^ ^33^ ^]^

SARS‐CoV‐2 infection has been shown to induce senescence in Vero cells^[^ ^34^ ^]^ and dopamine neurons^[^ ^35^ ^]^ in vitro. Senescence has also been observed in lung alveolar cells following SARS‐CoV‐2 infection,^[^ ^34^ ^]^ as well as in 16HBE bronchial epithelial cells.^[^ ^36^ ^]^ Using immuno‐lung organoid models, we found that SARS‐CoV‐2 infection triggers proinflammatory macrophage activation, leading to lung epithelial cell senescence. Further cell‐cell interaction analysis suggested that proinflammatory macrophages induces lung epithelial cell senescence through the THBS1‐(ITGA3+ITGB1) pathway. THBS1 encodes thrombospondin 1, a glycoprotein that is released and elevated during the acute phase of inflammation, playing a synergistic role in the inflammatory process. THBS1 was identified to be significantly upregulated in lung samples from 18 patients with fatal COVID‐19.^[^ ^37^ ^]^ Previous studies also associated THBS1 with senescence and SASP.^[^ ^38^ ^]^ Senescence is an emerging host response to various viral infections, not limited to SARS‐CoV‐2. Several viruses, including influenza A,^[^ ^39^ ^]^ HIV,^[^ ^40^ ^]^ CMV,^[^ ^41^ ^]^ have been shown to induce senescence in epithelial and immune cells. These findings support our observation of macrophage‐mediated senescence upon SARS‐CoV‐2 infection.

We successfully established hPSC‐derived immuno‐lung organoid models to investigate macrophage‐mediated host damage and uncover mechanisms underlying lung diseases. These advanced models are versatile tools for studying a range of infections, including SARS‐CoV‐2, influenza, and bacterial pathogens such as *Mycobacterium tuberculosis* (Mtb) and *Mycobacterium abscessus* (Mabs). They also provide valuable insights into non‐infectious conditions like pulmonary fibrosis, where macrophages are key contributors to tissue remodeling and disease progression. Furthermore, these organoids enable the study of lung regeneration, as anti‐inflammatory macrophages play essential roles in promoting tissue repair. By facilitating the exploration of macrophage‐lung cell interactions in disease and regeneration, immuno‐lung organoids serve as a powerful platform for understanding disease mechanisms, conducting drug screening, and advancing therapeutic development.

### 3.1 Limitation of the Study

The current CosMx study includes only 12–24 FOVs per sample, totaling 400 FOVs across 22 samples. We made a concerted effort to select anatomically comparable regions across non‐COVID, COVID‐A, and COVID‐E samples to enable meaningful comparisons. However, future studies with larger datasets and matched clinical criteria will be needed to enable more comprehensive and statistically robust analyses. In our study, we utilized the 1000‐plex RNA panel, which constrains our ability to perform comprehensive gene set enrichment analysis. We did not detect specific expression of HT2‐280 at the polarized ridge, which may reflect the immature nature of the hPSC‐derived alveolar cells. In the immuno‐lung organoid experiments, 293T cells were used to normalize the MOI,^[^ ^42^ ^]^ consistent with previous publications. However, we cannot entirely rule out the possibility that the 293T cell secretome may have influenced the results. Previous studies have reported significant alterations in myeloid cell populations in lung tissues from COVID‐19 patients, including distinct changes in alveolar macrophage subtypes.^[^ ^43^ ^]^ In the current study, we focused on proinflammatory macrophages in our immuno‐lung organoid model. Future studies will require the development of additional protocols to generate and incorporate a broader spectrum of myeloid cell subtypes, enabling a more comprehensive investigation of their respective roles in COVID‐19–associated lung injury. The ability of hPSCs to replicate infection dynamics depends on organoid maturity and would ideally, wherever possible, be validated against primary tissue.

## 4 Experimental Section

### Human Studies

Human lung tissues from non‐COVID donors, COVID‐19 patients undergoing transplantation and COVID‐19 patients were obtained from the Department of Pathology at Columbia University Medical Center in compliance with a protocol approved by the Columbia University Institutional Review Board (IRB: AAAB2667). All experiments were performed in accordance with the approved protocol. The donors’ information was listed in Table S1 (Supporting Information).

### Analysis of CosMx Spatial Transcriptomic Data

Samples extracted from patients of non‐COVID19 subjects, explant and autopsy samples from COVID19 patients were prepared according to manufacturer specifications. Tissue sections were cut into 5µm slides and prepared as specified by NanoString Technologies. Samples were imaged with configuration A. The slides were then incubated in xylene overnight, after which coverslips were removed, and the slides were stained with H&E.

CosMx data was exported after AtoMx preprocessing (v.1.3.2) using the export function and analyzed in R with Seurat (v.5.1.0).^[^ ^44^ ^]^ We applied an outlier p‐value cutoff of 0.01 to flag unreliable negative probes and retained cells with a minimum of 5 counts and a minimum of 10 counts per FOV, along with a negative control probe quantile cutoff of 0.5 for quality control. Probe counts were normalized and SCT‐transformed.^[^ ^45^ ^]^ Principal components were calculated, and 20 neighbors were identified to create a UMAP and Leiden clusters^[^ ^46^ ^]^ at a resolution of 2.0. ScType^[^ ^47^ ^]^ was used for semi‐automatic cell type annotation, using cell markers from LungMAP (https://www.lungmap.net/).^[^ ^48^ ^]^ We further refined cell types by tuning annotations and merging clusters corresponding to the same cell type. Differentially expressed genes (DEGs) in each cell type were identified using the “FindMarkers” function, comparing with the control group with criteria *logfc.threshold* = 0.25, *min.pct* = 0.25, and *test.use* = “MAST.” Expression of genes related to pro‐inflammatory, senescence, SASP, and pro‐/anti‐fibrosis markers was visualized across groups with DotPlot. Images of each FOV with or without molecules were visualized using “ImageDimPlot,” zoomed according to coordinates. The quantification of macrophage, monocyte, neutrophils, and lymphocytes were performed according to the corresponding cell number in each FOV and compared among three groups. The quantification of IGFBP6+ cells was conducted using the number of cells which expressed IGFBP6 in the selected FOVs.

### hiPSC Culture and Differentiation of Alveolar and Airway Organoids

All hPSC studies have been approved by the TRI‐SCI ESCRO committee. hPSCs were cultured on Matrigel‐coated plates in mTeSR1 medium (Stem Cell Technology) at 37 °C with 5% CO2. Airway organoids were differentiated following a previously published protocol.^[^ ^49^ ^]^ Briefly, hPSCs were dissociated into single cells with Gentle Cell Dissociation Reagent (Stem Cell Technologies, #07174) and plated (2 x 10^6 cells) in Matrigel‐coated 6‐well plates. After 24–48 h, the medium was changed to RPMI‐1640 supplemented with 1× Glutamax (Thermo Fisher Scientific), 50 µg mL^−1^ Normocin, 100 ng mL^−1^ Activin A (R&D systems), and 2 µM CHIR99021 (Cayman Chemical) for 24 h. Subsequently, cells were cultured in RPMI‐1640 medium supplemented with 1× Glutamax, 50 µg mL^−1^ Normocin, 0.2% fetal bovine serum (FBS, Corning), and 100 ng mL^−1^ Activin A for 2 days. Cells were then dissociated, passaged at a 1:3 or 1:4 ratio in DS/SB medium with 10 µM Y‐27632, and switched to DS/SB medium without Y‐27632 after 24 h. After 72 h of induction, cells were changed to CBRa medium, refreshed every 48 h. After 3‐5 days, CD47^hi^CD26^lo^ lung progenitor cells were sorted and resuspended at 1000 cells µL^−1^ in undiluted Matrigel, then replated in 50 µL drops in 24‐well plates. Epithelial organoids formed after several days and were passaged once the Matrigel drop was filled with cells. DS/SB and CBRa media were prepared following published protocols were sorted and resuspended at 1000 cells µL^−1^ in undiluted Matrigel, then replated in 50 µL drops in 24‐well plates. Epithelial organoids formed after several days and were passaged once the Matrigel drop was filled with cells. DS/SB and CBRa media were prepared following published protocols.^[^ ^49^ ^]^

### hPSC Differentiation to Macrophages

H9 hESCs expressing RFP (RFP‐H9) were differentiated following a previously reported protocol.^[^ ^50^ ^]^ RFP‐H9 cells were dissociated with ReLeSR (STEMCELL Technologies) and plated as small clusters onto Matrigel‐coated 6‐well plates at low density. After passaging, cells were cultured in IF9S medium with 50 ng mL^−1^ BMP‐4, 15 ng mL^−1^ Activin A, and 1.5 µM CHIR99021 for 2 days, followed by IF9S medium with 50 ng mL^−1^ VEGF, 50 ng mL^−1^ bFGF, 50 ng mL^−1^ SCF, and 10 µM SB431542. On days 5 and 7, the medium was refreshed with IF9S medium containing 50 ng mL^−1^ IL‐6, 10 ng mL^−1^ IL‐3, 50 ng mL^−1^ VEGF, 50 ng mL^−1^ bFGF, 50 ng mL^−1^ SCF, and 50 ng mL^−1^ TPO. On day 9, cells were dissociated with TrypLE and resuspended in IF9S medium with 50 ng mL^−1^ IL‐6, 10 ng mL^−1^ IL‐3, and 80 ng mL^−1^ M‐CSF. On day 13, the medium was changed to IF9S with IL‐6, IL‐3, and M‐CSF, and cells were harvested on day 15, plated on FBS‐coated plates in IF9S with M‐CSF. IF9S medium was prepared according to a previous publication RFP‐H9 cells were dissociated with ReLeSR (STEMCELL Technologies) and plated as small clusters onto Matrigel‐coated 6‐well plates at low density. After passaging, cells were cultured in IF9S medium with 50 ng mL^−1^ BMP‐4, 15 ng mL^−1^ Activin A, and 1.5 µM CHIR99021 for 2 days, followed by IF9S medium with 50 ng mL^−1^ VEGF, 50 ng mL^−1^ bFGF, 50 ng mL^−1^ SCF, and 10 µM SB431542. On days 5 and 7, the medium was refreshed with IF9S medium containing 50 ng mL^−1^ IL‐6, 10 ng mL^−1^ IL‐3, 50 ng mL^−1^ VEGF, 50 ng mL^−1^ bFGF, 50 ng mL^−1^ SCF, and 50 ng mL^−1^ TPO. On day 9, cells were dissociated with TrypLE and resuspended in IF9S medium with 50 ng mL^−1^ IL‐6, 10 ng mL^−1^ IL‐3, and 80 ng mL^−1^ M‐CSF. On day 13, the medium was changed to IF9S with IL‐6, IL‐3, and M‐CSF, and cells were harvested on day 15, plated on FBS‐coated plates in IF9S with M‐CSF. IF9S medium was prepared according to a previous publication.^[^ ^50^ ^]^ All differentiation steps were conducted under normoxic conditions at 37 °C, 5% CO2. hPSC‐derived macrophages were purified by magnetic sorting using anti‐CD14 beads. The macrophage was active to pro‐inflammatory state by stimulation with 100 ng mL^−1^ LPS and 20 ng mL^−1^ IFN‐γ for 48 h.

### Immuno‐Alveolar and Immuno‐Airway Organoids

Lung organoids and macrophages were derived as required. Optimal number of lung organoids and macrophages may vary depending on the cell line. The cells were briefly mixed using a 1000 µL pipette‐tip, where 1mm of the end of the tip was cut. The mix was then centrifuged at 300G for 3 min. Supernatant was removed as much as possible and cold Matrigel (Coring, 356231) was added. The tube was kept cold from this point on. Using a 1000ul‐pipette tip that is cold, organoids and cells were resuspended to small clusters. 50 µL per well in 24‐well plate. The plate was gently transferred to the incubator and allowed to polymerize for 20 min. The well was then filled with 1 mL of lung culture medium as described with 10uM Y‐27632. After 72 h, the medium was replaced with fresh medium. For some 2D image experiments, organoid clusters and macrophages were directly seeded on 1% Matrigel coated 96‐well plate.

### Immunhistochemistry

Cells were fixed in 4% PFA for 20 mins at room temperature, blocked with Mg^2+^/Ca^2+^‐free PBS containing 5% horse serum and 0.3% Triton‐X for 1 h, then incubated overnight at 4 °C with primary antibodies (**Extended Data** Table S2, Supporting Information). Secondary antibodies included donkey anti‐mouse, goat, rabbit, or chicken antibodies conjugated with Alexa‐Fluor‐488, Alexa‐Fluor‐594, or Alexa‐Fluor‐647 fluorophores (1:500, Life Technologies). Nuclei were counterstained with DAPI. Images were acquired on an LSM 880 Laser Scanning Confocal Microscope and processed with Zen software. Quantification was performed using ImageJ (NIH) software.

### SARS‐CoV‐2 Infection

SARS‐CoV‐2, isolate USA‐WA1/2020, was obtained from NIH (BEI Reagents Cat # NR‐52281). This virus was propagated in Vero E6 cells (ATCC) in EMEM supplemented with 10% FCS, 1 mM sodium pyruvate, and 10 mM HEPES, as previously described.^[^ ^51^ ^]^ Following propagation, the virus‐containing supernatant was harvested and aliquoted into sterile vials. These aliquots were then cryopreserved at ‐80 degrees Celsius for long‐term storage. The plaque‐forming units (PFU) were validated to ensure the viral titer's accuracy. For subsequent experiments, new vials were thawed from the freezer to infect cells, maintaining the consistency of viral infection efficiency across all studies. The serially diluted virus was added to the cells, then the cells were harvested and evaluated to determine the multiplicity of infection (MOI).

SARS‐CoV‐2 infections of hPSC‐derived lung organoids were conducted in culture media at the specified MOIs at 37 °C in a 5% CO2 incubator. At designated hours post‐infection (hpi), cells were washed three times with PBS. For RNA analysis, cells were lysed in TRIzol (Invitrogen). For multi‐omics RNAseq, cells underwent a treatment protocol before being loaded into the 10x Genomics Chromium Controller for subsequent library preparation and for immunofluorescence staining, cells were fixed in 4% formaldehyde for 60 mins at room temperature.

All work involving live SARS‐CoV‐2 was performed in the CDC/USDA‐approved BSL‐3 facility at the Aaron Diamond AIDS Research Center, Columbia University.

### Single‐Cell RNA‐seq Data Analysis of Macrophage‐Alveolar and Macrophage‐Airway Organoids— Sequencing and Pre‐Processing

The 10X libraries were sequenced on the Illumina NovaSeq6000 sequencer with paired‐end reads (28 bp for read 1 and 90 bp for read 2). Sequencing data were primarily analyzed using the 10X cellranger pipeline v7.1.0 in a two‐step process. First, cellranger *mkfastq* was used to demultiplex the samples and generate FASTQ files. Second, cellranger *count* aligned the FASTQ data to a customized reference genome, producing a gene expression UMI counts matrix for each library. The customized reference genome was constructed by integrating the 10X pre‐built human reference GRCh38‐2020‐A and the SARS‐CoV‐2 virus genome using the cellranger mkref. The SARS‐CoV‐2 genome was obtained from the NCBI Nucleotide database (accession number NC_045512.2).

### Cell Quality Control (QC)

Cells with fewer than 300 or more than 9,000 detected genes, fewer than 600 or more than 75,000 detected transcripts, or mitochondrial gene content exceeding 10% were excluded. Doublets were identified for each sample using the R DoubletFinder package^[^ ^52^ ^]^ v2.0.3, assuming a doublet rate 0.8% per 1,000 recovered cells as reported by 10X Genomics. Identified doublets were excluded from downstream analysis.

### Normalization and Integration

Normalization of gene expression UMI counts was performed using a deconvolution strategy implemented with the R scran^[^ ^53^ ^]^ (v.1.22.1), scuttle^[^ ^54^ ^]^ (v1.4.0) and batchelor^[^ ^55^ ^]^ (v1.10.0) packages. The workflow included pre‐clustering cells using the *quickCluster* function in scran, computing size factors per cell within each cluster, rescaling these factors across clusters, normalizing UMI counts per cell by the size factors using the *computeSumFactors* function in scran, and applying a logarithmic transformation with the *logNormCounts* function in scuttle. Normalization across samples was performed using the *multiBatchNorm* function in the batchelor. Cells from multiple samples were aligned using the *quickCorrect* function in batchelor, which involved identifying highly variable genes, performing principle component analysis (PCA) on those genes and correcting PCs based on their mutual nearest neighbors (MNNs). The top 50 corrected PCs were retained for downstream clustering.

### Clustering and Visualization

UMAP dimensional reduction was performed using the *RunUMAP* function in the R Seurat package^[^ ^56^ ^]^ v4.1.0, with the number of neighboring points set to 30 and the training epochs set to 500. Clustering was conducted by constructing a shared nearest neighbor (SNN) graph and grouping cells with similar transcriptomic profiles using the *FindNeighbors* and *FindClusters* functions in Seurat. For macrophage‐alveolar organoids, clustering was performed at a resolution of 0.4, resulting in twenty clusters that, after reviewing the clusters, were merged into eight clusters representing AT2 cells, AT1 cells, Basal cells –1, Basal cells‐2, Basal cells‐3, Macrophages, Fibroblasts and 293T cells. For macrophage‐airway organoids, clustering was performed at a resolution of 0.8, resulting in twenty clusters that were merged into seven clusters: Basal cells, Ciliated cells, Tuft cells, Virus‐infected cells, Macrophages, Fibroblasts, and 293T cells.

UMAP plots were generated using the R ggplot2 package^[^ ^57^ ^]^ v3.4.1 to visualize cell clusters and marker gene expressions. Dot plots were generated using the *DotPlot* function in Seurat to depict gene expression changes across conditions.

To evaluate the cell fates of hPSC‐derived airway and alveolar cells, we curated marker genes representing fifteen reference cell types from the human adult lung, as reported by a previous study.^[^ ^58^ ^]^ We assessed similarity by calculating the fraction of overlapping marker genes between our clusters and the reference cell types. The resulting overlap fractions were standardized using z‐score transformation across reference cell types. A heatmap illustrating cell‐type similarity was generated using the R pheatmap package.

### Differential Expression (DE) and Gene Set Enrichment Analysis

DE analysis was performed on AT2 cells between alveolar organoids exposed to SARS‐CoV‐2 and alveolar organoids co‐cultured with 293T cells exposed to SARS‐CoV‐2, using the Wilcoxon rank‐sum test via the *FindMarkers* function in Seurat. Genes were ranked by the average log2 fold change and gene set enrichment analysis (GSEA) was conducted on cell death pathways using the *GSEA* function in the R clusterProfiler package^[^ ^59^ ^]^ v4.6.2. Results were visualized with bar plot using R ggplot2^[^ ^57^ ^]^ v3.4.1, and enrichment plot for the senescence pathway was generated with the *gseaplot2* function in the R enrichplot package^[^ ^60^ ^]^ v1.18.3.

### Cell–Cell Interaction Analysis

To investigate the mechanisms by which proinflammatory macrophages induce lung cell senescence, cell–cell interaction analysis was performed using the R CellChat package^[^ ^61^ ^]^ v2.1.2. For macrophage‐alveolar organoids, interactions between macrophages and AT2 cells were analyzed, while for macrophage‐airway organoids, interactions between macrophages and ciliated cells were examined. The tiny 293T cell clusters, observed only in 293T co‐cultured samples, were excluded from the analysis. Communication probabilities mediated by ligand‐receptor pairs in between macrophages and AT2 cells (for macrophage‐alveolar organoids) and in between macrophages and ciliated cells (for macrophage‐airway organoids) were visualized using bubble plots generated with the *netVisual_bubble* function in CellChat.

### Single‐Nucleus Multi‐Omics Analysis of Immuno‐Alveolar and Immuno‐Airway Organoids—Sequencing and Pre‐Processing

The 10X Single Cell Multiome ATAC + Gene Expression (GEX) libraries were sequenced on the Illumina NovaSeqXplus sequencer with paired‐end reads (28 bp for read 1 and 91 bp for read 2 for GEX libraries, 51 bp for read 1, 24 bp for read 2 and 51 bp for read 3 for ATAC libraries). Sequencing data were primarily analyzed using the 10X cellranger‐arc pipeline v2.0.1 in a two‐step process. First, cellranger‐arc mkfastq was used to demultiplex the samples and generate FASTQ files. Second, cellranger‐arc count aligned the FASTQ data to the 10X pre‐built GRCh38‐2020‐A‐2.0.0 human reference genome, producing a gene expression UMI counts matrix for each GEX library and peak and fragment files for each ATAC library.

### Cell Quality Control (QC)

For GEX libraries, cells with fewer than 300 or more than 7,000 detected genes, fewer than 600 or more than 30,000 detected transcripts, or mitochondrial gene content exceeding 20% were excluded. Doublets were identified for each sample using the R DoubletFinder package^[^ ^52^ ^]^ v2.0.3, assuming a doublet rate 0.8% per 1,000 recovered cells as reported by 10X Genomics, and excluded from downstream analysis.

For ATAC libraries, a common set of peaks across all samples was created using the *reduce* function. Peaks larger than 10,000 bp or smaller than 20 bp were excluded. A peaks x cells matrix was generated for each sample by quantifying the common peaks using the *FeatureMatrix* function in R Signac^[^ ^62^ ^]^ package v1.10.0. Cells were excluded if they had fewer than 700 or more than 25,000 detected peaks, fewer than 50% of reads in peaks, more than 1% of reads in blacklist regions, a mononucleosomal‐to‐nucleosome‐free fragments ratio greater than 1, or TSS enrichment score less than 3. Cells passing both GEX and ATAC QC criteria were used for downstream analysis.

### Normalization and Integration

For GEX libraries, normalization was performed using a deconvolution strategy implemented with the R scran^[^ ^53^ ^]^ (v.1.22.1), scuttle^[^ ^54^ ^]^ (v1.4.0) and batchelor^[^ ^55^ ^]^ (v1.10.0) packages. The workflow included pre‐clustering cells using the *quickCluster* function in scran, computing size factors per cell within each cluster, rescaling these factors across clusters, normalizing UMI counts per cell by the size factors using the *computeSumFactors* function in scran, and applying a logarithmic transformation with the *logNormCounts* function in scuttle. Normalization across samples was performed using the *multiBatchNorm* function in the batchelor. Cells from multiple samples were aligned using the *quickCorrect* function in batchelor, which involved identifying highly variable genes, performing PCA on those genes and correcting PCs based on their MNNs. The top 50 corrected PCs were retained for clustering.

For ATAC libraries, normalization was performed using the R Signac package^[^ ^62^ ^]^ v1.10.0. Peaks were ranked and selected using the *FindTopFeatures* function, and term frequency‐inverse document frequency (TF‐IDF) normalization was performed using the *RunTFIDF* function. Latent semantic indexing (LSI) components were computed using the *RunSVD* function, with the top 50 LSI components (excluding the first) retained for clustering. Cells from multiple samples were aligned by finding integration anchors among samples with the *FindIntegrationAnchors* function and integrating low‐dimensional embeddings across samples using the *IntegrateEmbeddings* function.

### Clustering and Visualization

For GEX data, clustering was conducted by constructing a SNN graph and grouping cells with similar transcriptomic profiles using the *FindNeighbors* and *FindClusters* functions in Seurat. For immuno‐alveolar organoids, clustering was performed at a resolution of 0.65, resulting in fourteen clusters that, after review, were merged into eight clusters representing AT2 cells, AT1 cells, Basal cells –1, Basal cells‐2, Basal cells‐3, Macrophages, Fibroblasts. For immuno‐airway organoids, clustering was performed at a resolution of 0.7, resulting in thirteen clusters that were merged into five clusters: Ciliated cells, Basal cells‐1, Basal cells‐2, Fibroblasts, and Macrophages.

For both GEX and ATAC data, UMAP dimensional reduction was performed using either the corrected PCs or integrated embeddings with the *RunUMAP* function in the R Seurat package^[^ ^56^ ^]^ v4.1.0, with the number of neighboring points set to 30 and the training epochs set to 500. Joint UMAP visualization combining GEX and ATAC modalities was constructed using the *FindMultiModalNeighbors* function in Seurat to create a weighted nearest neighbor (WNN) graph, followed by dimensional reduction with the *RunUMAP* function.

UMAP plots were generated using the R ggplot2 package^[^ ^57^ ^]^ v3.4.1 to visualize cell clusters and marker gene expressions. Dot plots were generated using the *DotPlot* function in Seurat to depict gene expression changes across conditions.

### Cell–Cell Interaction Analysis

To investigate the mechanisms by which proinflammatory macrophages induce lung cell senescence, cell‐cell interaction analysis was performed using the R CellChat package^[^ ^61^ ^]^ v2.1.2. For immuno‐alveolar organoids, interactions between macrophages and AT2 cells were analyzed. For immuno‐airway organoids, interactions between macrophages and ciliated cells were examined. Communication probabilities mediated by ligand‐receptor pairs in between macrophages and AT2 cells (for immuno‐alveolar organoids) and in between macrophages and ciliated cells (for immuno‐airway organoids) were visualized using bubble plots generated with the *netVisual_bubble* function in CellChat.

### Electron Microscopy

Alveolospheres were washed with PBS and fixed in primary fix buffer (2.5% glutaraldehyde, 4% Paraformaldehyde, 0.002% picric acid in 0.1M sodium cacodylate) for 30 min at room temperature. Samples were washed three times in 0.1M sodium cacodylate 20 min each. Post‐fixation was performed using osmium (1.5% Osmium tetroxide, 3% K‐ferricyanide aqueous solution) for 90 min, followed by three additional washes in 0.1M sodium cacodylate 20 min each. Then samples were block stained in 1.5% Uranyl acetate for 30 min, dehydrated through graded ethanol series, and transitioned through acetonitrile. The samples were embedded in Embed 812 resin (Electron Microscopy Sciences, Hatfield, PA) and polymerized overnight. Ultrathin sections (70 nm, silver‐gold thickness) were cut using a Diatome diamond knife (Diatome, USA) on a Leica Ultracut S ultramicrotome. Sections were mounted on grids and stained with 1.5% aqueous uranyl acetate for 15 min at 60 °C, followed by Lead Citrate for 5 min at room temperature. Sections were examined using a JEM 1400 transmission electron microscope (JEOL USA, Inc., Peabody, MA) operated at 80 kV. Digital images were captured with a Veleta 2K × 2K camera (Olympus Soft Imaging Solutions, GmbH).

### Website

In this approach, a Large Language Model (LLM)‐based agent, ChatGPT‐4o, was utilized as an AI assistant to facilitate user interaction with LungSpatialDB. This assistant processes users' natural language queries through sophisticated prompt engineering, enabling it to interpret and respond effectively to diverse questions. The assistant analyzes the input query, identifies its intent, and dynamically decides whether to invoke backend APIs, such as retrieving figure results from LungSpatialDB. For example, for a query like “How about the markers’ expression of macrophage?” the assistant first leverages its embedded knowledge and prompt design to identify marker genes associated with macrophages and then invokes the backend API to present the corresponding results. This functionality is achieved through seamless integration of LLM capabilities with our API infrastructure, ensuring accurate and context‐relevant outputs. The assistant is designed to handle multiple queries concurrently, making it highly efficient in responding to diverse user requests. By combining the immense knowledge embedded in ChatGPT‐4o with advanced prompt design, the system enhances the accessibility and utility of LungSpatialDB for a wide range of biological research applications.

### Statistical Analysis

N = 3 independent biological replicates were used for all experiments unless otherwise indicated. Normalization calculation between the experimental group and the control group. *P*‐values were calculated by unpaired two‐tailed Student's t‐test or one way ANOVA with a common control unless otherwise indicated. n.s. indicates a non‐significant difference. *p < 0.05, **p < 0.01 and ***p < 0.001. Statistical analysis of teratoma size measurements was performed in the GraphPad Prism software (version 9.5.1).

## Conflict of Interest

S.C. and T.E. are the co‐founders of Oncobeat, Inc. S.C is the co‐founder of iOrganBio, Inc. The other authors have no conflict of interest.

## Author Contributions

Y.H., D.L., T.Z., and J.G. contributed equally to this work. S.C., J.Q., Y.H., T.E., and L.Y. conceived the study and designed the experiments. Y.H., L.Y., T.L., X.D., N.S., and Z.M. performed organoids differentiation, macrophage differentiation, co‐culture assay and immunostaining assays. D.L. and T.Z. performed data analysis. J.Z.X., C.P., and Y.S. helped with 10x libraries preparation. J.G. and M.S.N. assisted the SARS‐CoV‐2 preparation and infection. Z.H., T.J., Y.H., M.Z., and J.L. assisted the website. Y.F., A.S., and H.H. helped the human tissue slides preparation. S.C., Y.H., L.Y., and D.L. wrote the manuscript.

## Supporting information



Supporting Information

## Data Availability

All data are available in the main text or the supplementary materials. The raw sequence data are uploaded to the GEO database (GSE287114).
