# Supplementary material for: A Human Immuno‐Lung Organoid Model to Study Macrophage‐Mediated Lung Cell Senescence Upon SARS‐CoV‐2 Infection
Source: Adv Sci (Weinh). 2025 Jul 25;12(36):e03932. doi: 10.1002/advs.202503932 (PMC12463091; doi:10.1002/advs.202503932)
Supplement: Supplementary file 1 — Supporting Information [file ADVS-12-e03932-s001.pdf]

ADVANCED SCIENCE
Open Access

Supporting Information

for *Adv. Sci.*, DOI 10.1002/advs.202503932

A Human Immuno-Lung Organoid Model to Study Macrophage-Mediated Lung Cell Senescence Upon SARS-CoV-2 Infection

Yuling Han, Dongliang Leng, Tuo Zhang, Jian Ge, Yinshan Fang, Tiankun Lu, Xue Dong, Manoj S Nair, Neranjan de Silva, Zhaowei Han, Tiancheng Jiao, Yuanhao Huang, Meiqi Zhao, Anjali Saqi, Hanina Hibshoosh, Zihe Meng, Jenny Z Xiang, Chendong Pan, Yanjie Sun, David D. Ho, Todd Evans, Jie Liu, Liuliu Yang*, Jianwen Que* and Shuibing Chen**

Title: A Human Immuno-Lung Organoid Model to Study Macrophage-Mediated Lung Cell Senescence Upon SARS-CoV-2 Infection.

Authors: Yuling Han[1,2,#, *], Dongliang Leng[1,2,#,], Tuo Zhang[3,#], Jian Ge[4, #], Yinshan Fang[4], Tiankun Lu[1,2], Xue Dong[1], Manoj S Nair[5], Neranjan de Silva[1,2], Zhaowei Han[6], Tiancheng Jiao[6], Yuanhao Huang[6], Meiqi Zhao[6], Anjali Saqi[7], Hanina Hibshoosh[7], Zihe Meng[1,2], Jenny Z Xiang[3], Chendong Pan[3], Yanjie Sun[3], David D. Ho[5], Todd Evans[1,2], Jie Liu[6], Liuliu Yang[1,2,*,], Jianwen Que[6,*], Shuibing Chen[1,2,8, *]

Extended Data Figure 1

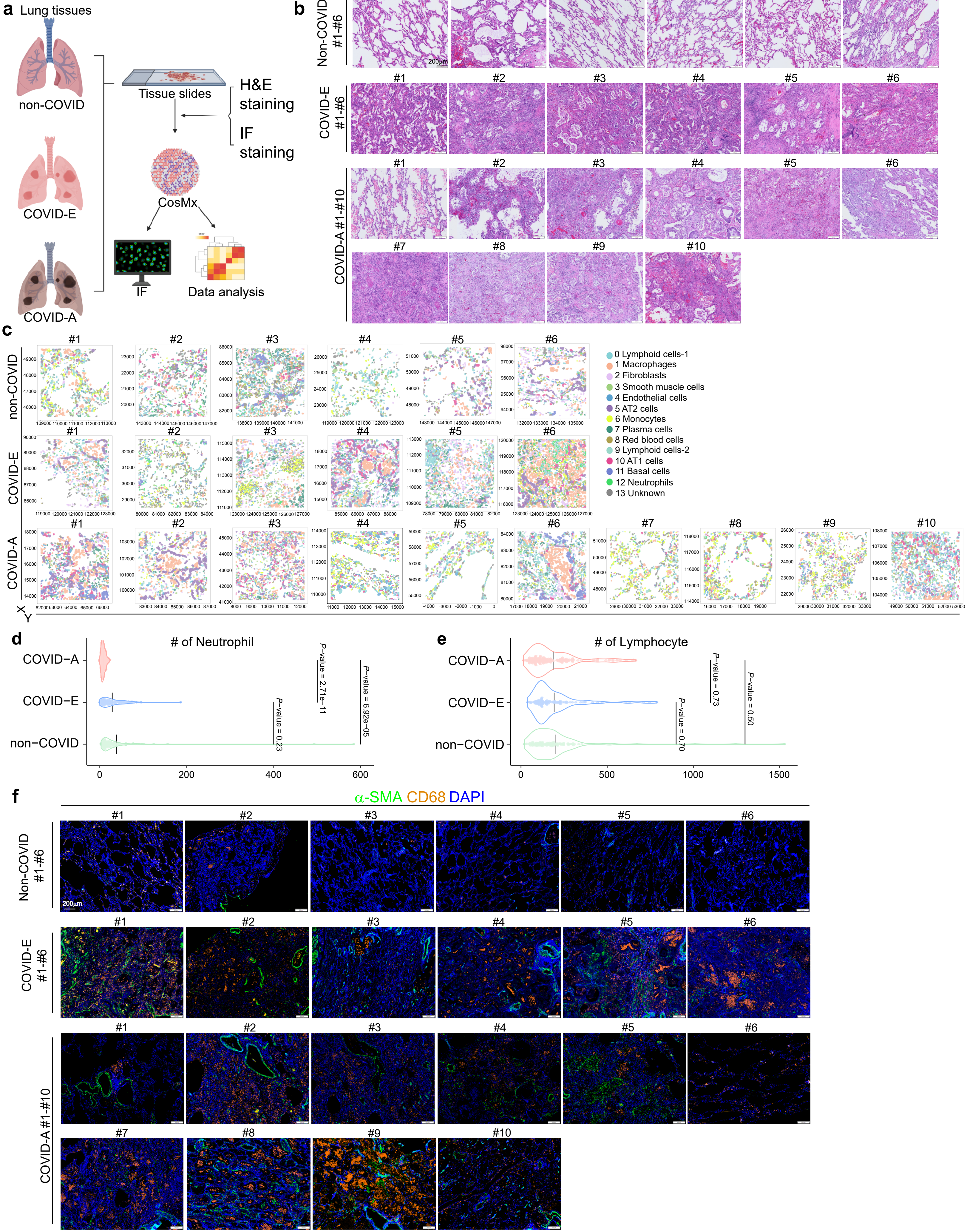

Extended Data Fig. 1. Spatial transcriptomics analysis of lung explant and autopsy samples from COVID-19 patients.

a, Schematic representation of CosMx spatial transcriptomics and protein analysis. **b,** H&E staining of human lung tissues of non-COVID (N=6), COVID-E (N=6), COVID-A (N=10) subjects. Scale bar=200 μm. **c,** Representative images plots showed the enrichment of macrophages in human lung samples of non-COVID (N=6), COVID-E (N=6), COVID-A (N=10) subjects. **d, e,** Bar plot showed the percentage of lymphoid cells and neutrophils of human lung samples of non-COVID (N=6), COVID-E (N=6), COVID-A (N=10) subjects. **f,** Representative images of immunostaining of CD68 and a-SMA of human lung samples of non-COVID (N=6), COVID-E (N=6), COVID-A (N=10) subjects. Scale bar=200 μm.

Extended Data Figure 2

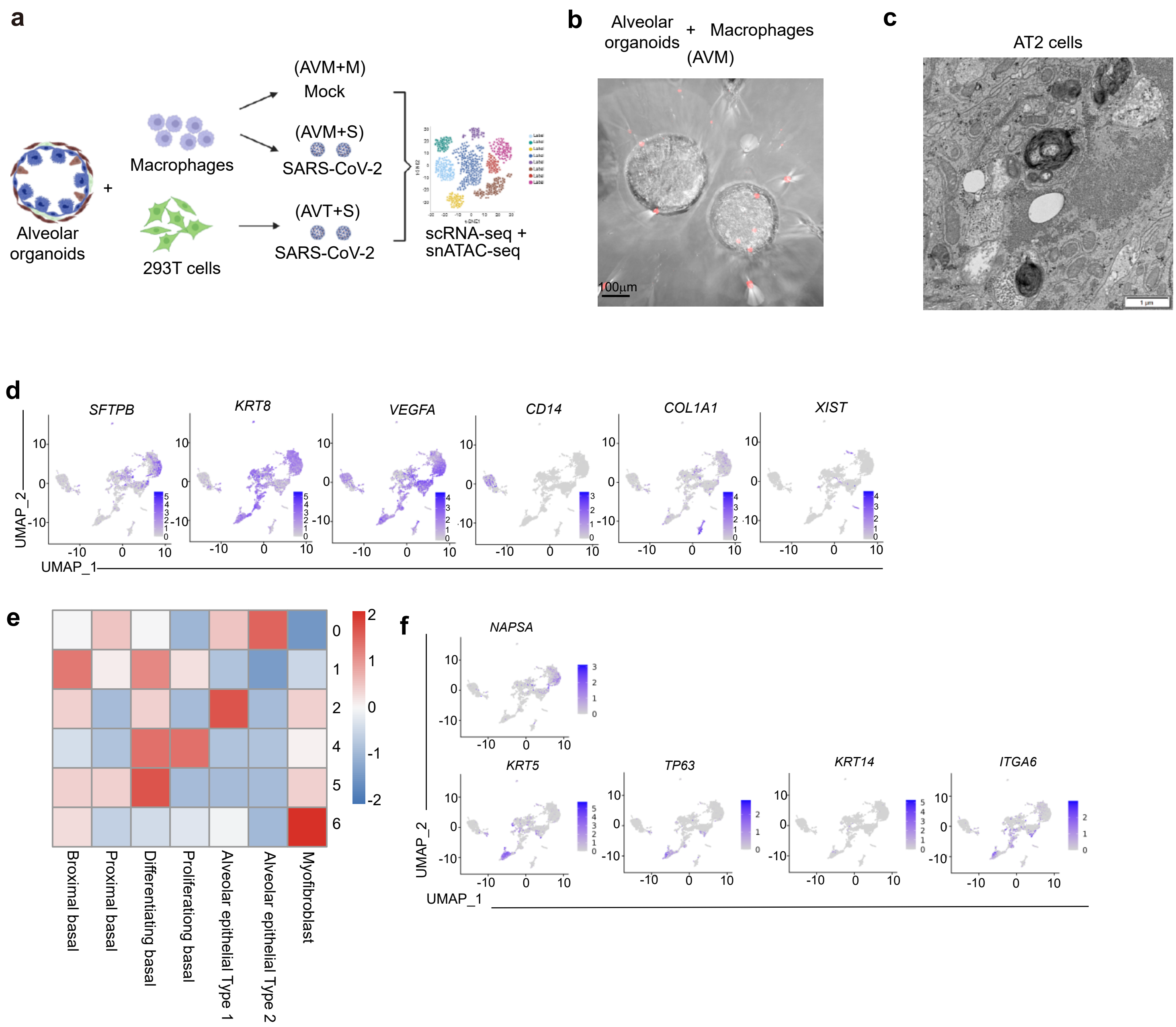

Extended Data Fig. 2. Characterization of hPSC-derived immuno-alveolar organoids.

a, Schematic representation of hPSC-derived immuno-alveolar organoids. **b,** Bright field and fluorescence images of hPSC-derived immuno-alveolar organoids. **c,** Electron microscopy analysis of the alveolar organoids. Scale bar= 200 nM. **d,** UMAP of marker gene for each cluster of immuno-alveolar organoids. **e,** Correlation analysis of genes with cell fates in hPSC-derived immuno-alveolar organoids and adult human lung cells. **f,** UMAP of additional marker genes for each cluster of immuno-alveolar organoids.

Extended Data Figure 3

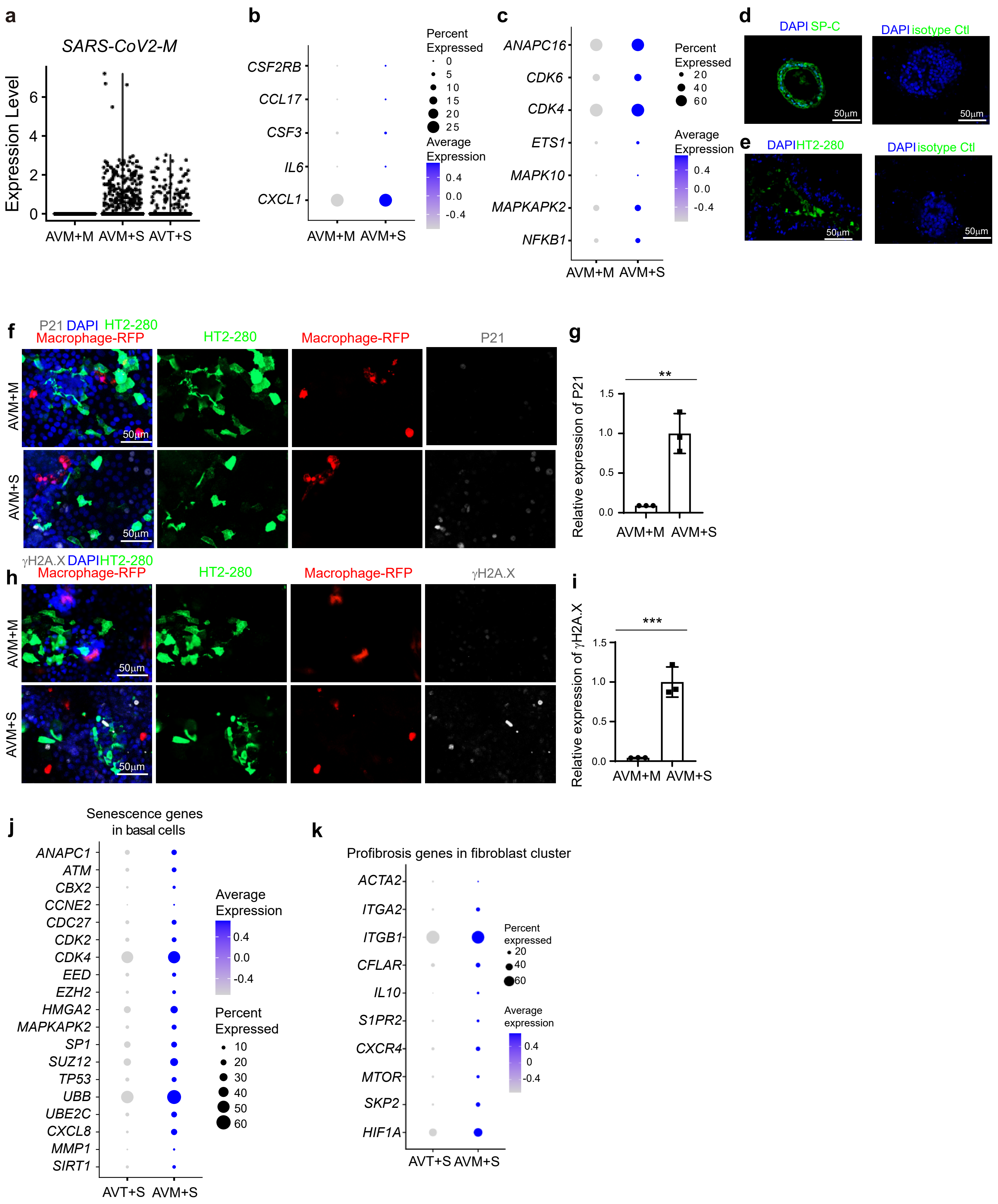

Extended Data Fig. 3. hPSC-derived immuno-alveolar organoids to model macrophage-mediated lung cell damage.

a, Dot plot showing the detection of SARS-CoV2-M transcript hPSC-derived immuno-alveolar organoids exposed to mock (AVM+M) or SARS-CoV-2 (MOI=0.25, AVM+S), and alveolar organoids co-cultured with 293T cells exposed to SARS-CoV-2 (MOI=0.25, AVT+S). **b**, Dot plot analysis of SASP associated genes in AT2 cell cluster of AVM+S and AVM+M conditions. **c,** Dot plot analysis of senescence associate genes in AT2 cell cluster of AVM+S and AVM+M conditions. **d, e,** Isotype controls of immunostaining using antibodies against pro-SP-C (d) and HT2-280. Scale bar = 50 µm. **f, g,** Immunostaining (f) and quantification (g) of the relative expression of p21 in of AVM+S and AVM+M conditions. Scale bar= 50 µm. **h, i,** Immunostaining (h) and quantification (i) of the relative expression of gH2A.X in of AVM+S and AVM+M conditions. Scale bar= 50 µm. **j,** Dot plot analysis of senescence associated genes in basal cells of AVM+S and AVM+M conditions. **k,** Dot plot analysis of fibrosis associated genes in fibroblast cluster of AVM+S and AVT+S conditions.

Extended Data Figure 4

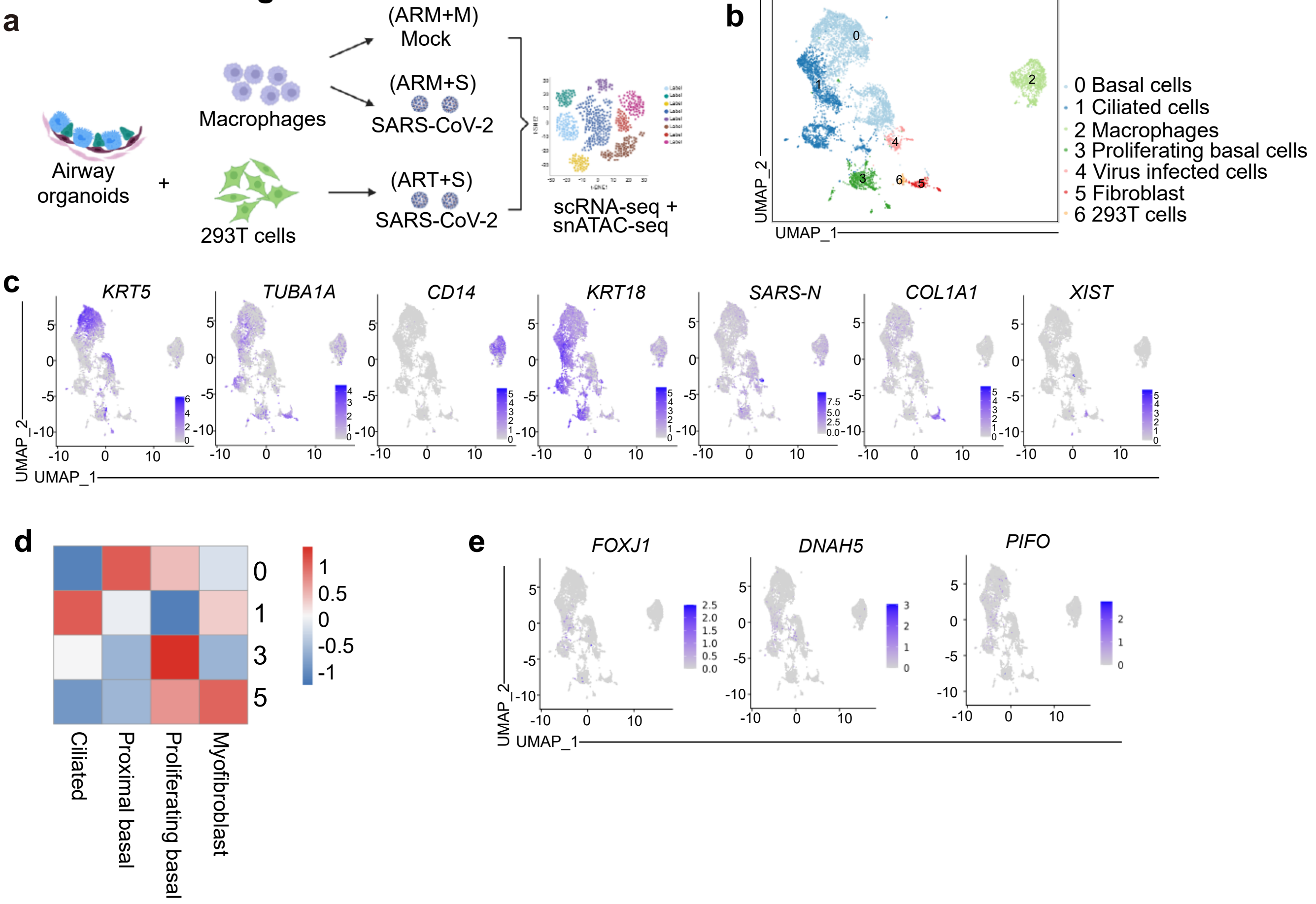

Extended Data Fig. 4. Characterization of hPSC-derived immuno-airway organoids. **a,** Schematic representation of hPSC-derived immuno-airway organoids construction and infected with mock or SARS-CoV-2 (MOI=0.05). **b,** UMAP of marker gene for each cluster of hPSC-derived immuno-airway organoids. **c,** UMAP of marker gene for each cluster of immuno-airway organoids. **d,** Correlation analysis of genes with cell fates in hPSC-derived immuno-airway organoids and adult human lung cells. **e,** UMAP of additional marker genes for each cluster of immuno-airway organoids.

Extended Data Figure 5

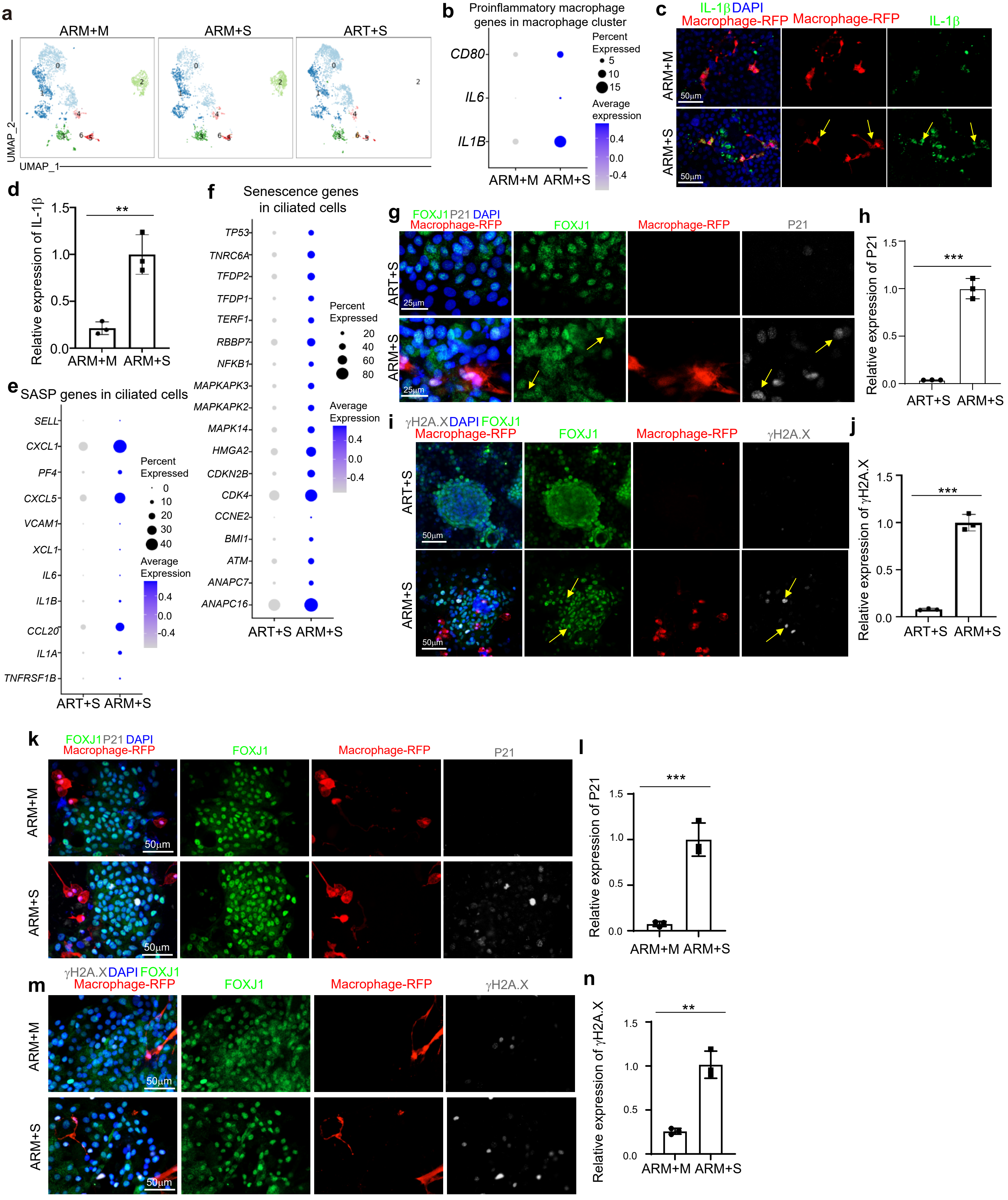

Extended Data Fig. 5. hPSC-derived immuno-airway organoids to model macrophage-mediated lung cell damage upon SARS-CoV-2 infection.
a, Individual UMAP of immuno-airway organoids exposed to mock (ARM+M) or SARS-CoV-2 (MOI=0.05, ARM+S), and airway organoids co-cultured with 293T cells exposed to SARS-CoV-2 (MOI=0.05, ART+S). **e,** Dot plot analysis of proinflammatory macrophage associated genes in macrophage cluster of ARM+M or ARM+S conditions. **c, d,** Immunohistochemistry staining (c) and quantification (d) of the relative expression of IL-1β in RFP^+ macrophages of ARM+M or ARM+S conditions. The yellow arrows highlight the expression of IL-1β in RFP^+ macrophages. Scale bar= 50 μm. **e,** Dot plot analysis of SASP associate genes in Ciliated cell cluster of ARM+S and ART+S conditions. **f,** Dot plot analysis of senescence associate genes in Ciliated cell cluster of macrophage-airway organoids of ARM+S and ART+S conditions. **g, h,** Immunostaining (g) and quantification (h) of the relative expression of p21 in FOXJ1+ ciliated cells of ARM+S and ART+S conditions. The yellow arrows highlight the expression of p21 in FOXJ1+ Ciliated cells. Scale bar= 50 μm. **i, j,** Immunostaining (i) and quantification (j) of the relative expression of γH2A.X in FOXJ1+ ciliated cells of ARM+S and ART+S conditions. The yellow arrows highlight the expression of γH2A.X in FOXJ1+ Ciliated cells. Scale bar= 50 μm. **k, l,** Immunostaining (k) and quantification (l) of the relative expression of p21 in FOXJ1+ ciliated cells of ARM+S and ARM+M conditions. The yellow arrows highlight the expression of p21 in FOXJ1+ Ciliated cells. Scale bar= 50 μm. **m, n,** Immunostaining (m) and quantification (n) of the relative expression of γH2A.X in FOXJ1+ ciliated cells of ARM+S and ARM+M conditions. The yellow arrows highlight the expression of γH2A.X in FOXJ1+ Ciliated cells. Scale bar= 50 μm. N=3 independent biological replicates. Data was presented as mean ± STDEV. *P* values were calculated by unpaired two-tailed Student's t test. **$P < 0.01$, ***$P < 0.001$.

Extended Data Figure 6

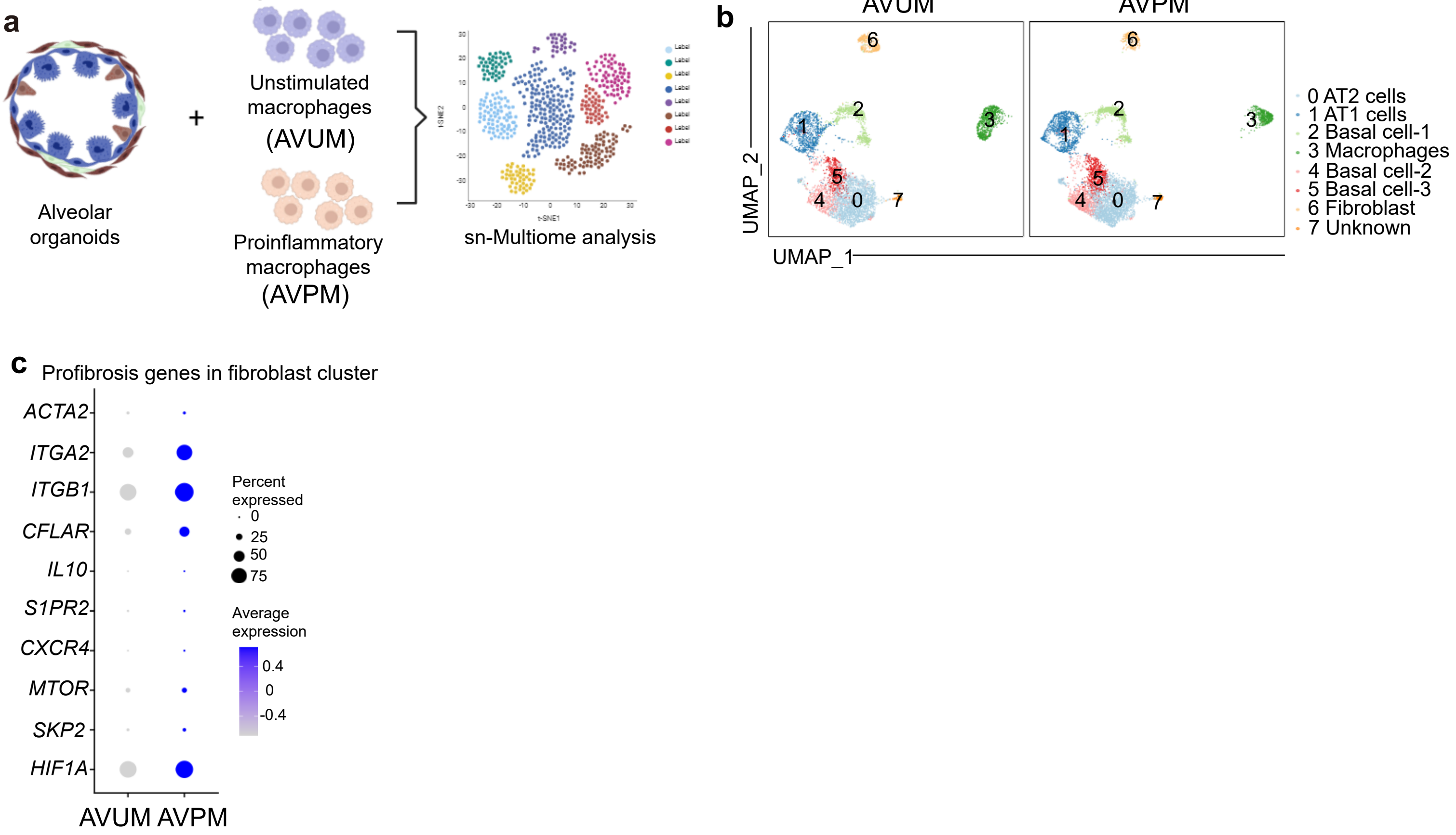

Extended Data Fig. 6. Construction and sn-multiomics analysis of alveolar organoids containing unstimulated and proinflammatory macrophages.

a, Schematic representation of hPSC-derived alveolar organoids containing unstimulated (AVUM) and proinflammatory (AVPM) macrophages. **b,** Individual UMAP of snRNA-seq and snATAC-seq of AVUM and AVPM macrophages. **c,** Dot plot analysis of fibrosis associated genes in fibroblast cluster of ARM+S and ART+S conditions.

Extended Data Figure 7

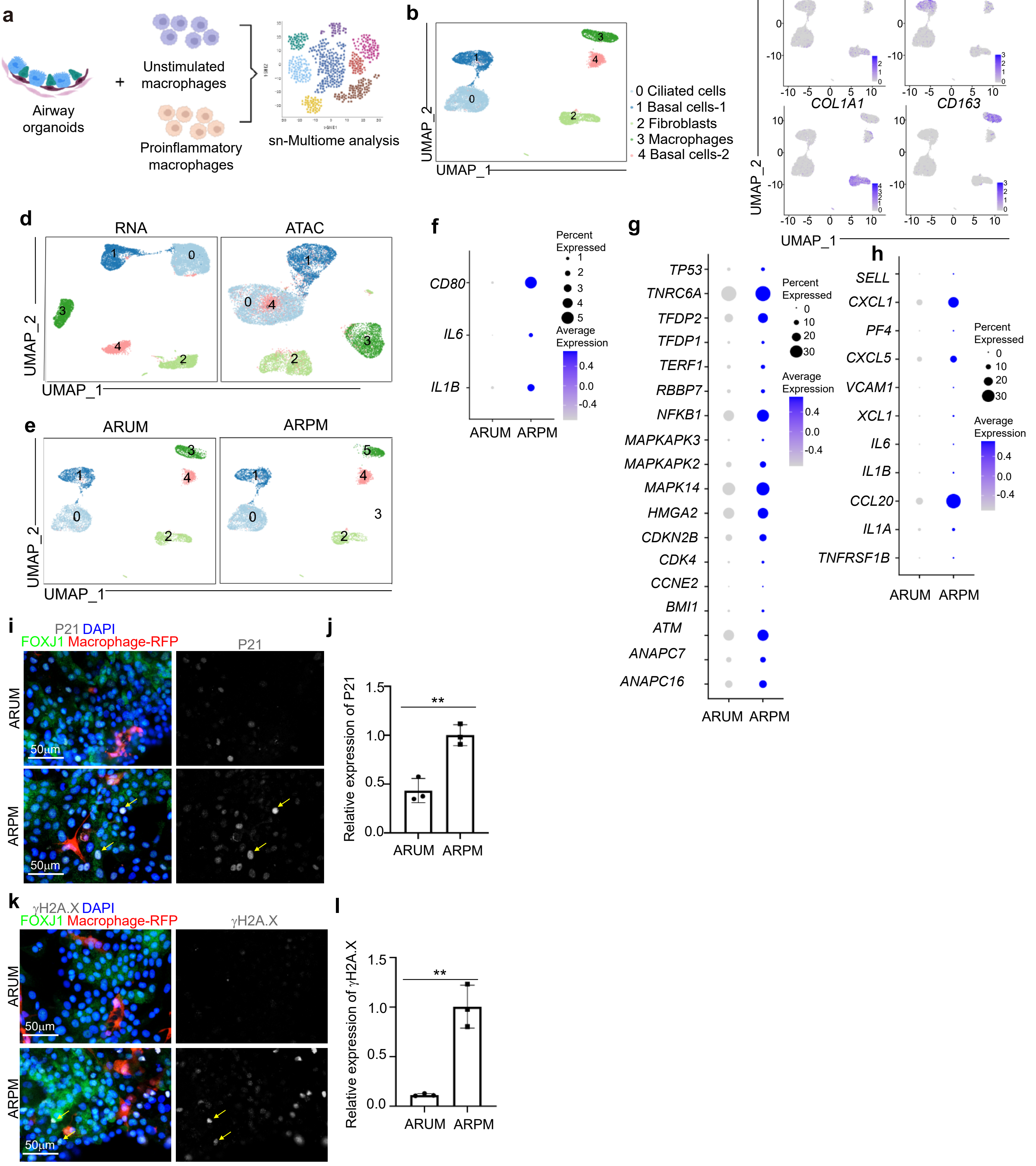

Extended Data Fig. 7. Construction and sn-multiomics analysis of airway organoids containing unstimulated and proinflammatory macrophages.

a, Schematic representation of hPSC-derived lung airway organoids containing unstimulated (ARUM) and proinflammatory (ARPM) organoids. **b,** UMAP of ARUM and ARPM organoids. **c,** UMAP of marker gene for each cluster of hPSC-derived immuno-airway organoids. **d,** Individual UMAP of snRNA-seq and snATAC-seq analysis. **e,** Individual UMAP of scRNA-seq analysis of ARUM and ARPM organoids. **f,** Dot plot analysis of proinflammatory macrophage associated genes in ciliated cell cluster of ARUM and ARPM organoids. **g,** Dot plot analysis of SASP associate genes in ciliated cell cluster of ARUM and ARPM organoids. **h,** Dot plot analysis of senescence associated genes in ciliated cell cluster of ARUM and ARPM organoids. **i, j,** Immunostaining (i) and quantification (j) of the relative expression of p21 in ARUM and ARPM organoids. The yellow arrows highlight the expression of p21 in FOXJ1+ Ciliated cells. Scale bar= 50 μm. **k, l,** Immunostaining (k) and quantification (l) of the relative expression of γH2A.X in ARUM and ARPM organoids. The yellow arrows highlight the expression of γH2A.X in FOXJ1+ ciliated cells. Scale bar= 50 μm. N=3 independent biological replicates. Data was presented as mean ± STDEV. *P* values were calculated by unpaired two-tailed Student's t test. $^{**}P < 0.01$.

Extended Data Figure 8

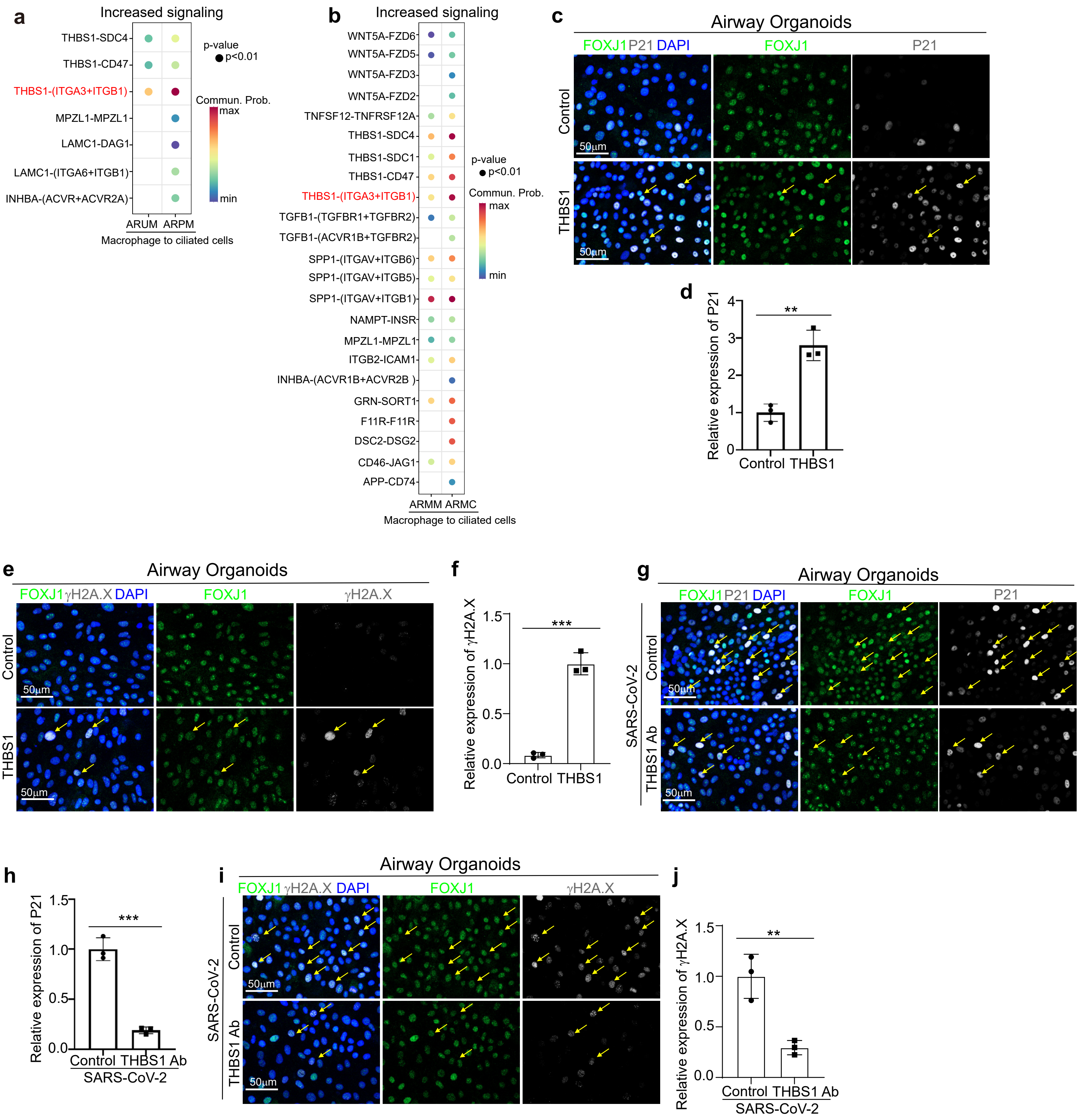

Extended Data Fig. 8. THBS1-(ITGA3+ITGB1) contributes to proinflammatory macrophage-mediated lung cell senescence.

a, Dot plot showing the differential signaling from macrophages to ciliated cells in ARUM and ARPM organoids. **b,** Dot plot showing the differential signaling from macrophages to ciliated cells in hPSC-derived ARM+M and ARM+S conditions. **c, d,** Immunostaining (c) and quantification (d) of the relative expression of p21 in hPSC-derived airway organoids treated with control or 5 mg/ml THBS1 protein. The yellow arrows highlight the expression of p21 in FOXJ1$^+$ ciliated cells. Scale bar= 50 μm. **e, f,** Immunostaining (e) and quantification (f) of the relative expression of γH2A.X in hPSC-derived airway organoids treated with control or 5mg/ml THBS1 protein. The yellow arrows highlight the expression of gH2A.X in FOXJ1$^+$ ciliated cells. Scale bar= 50 μm. **g, h,** Immunostaining (g) and quantification (h) of the relative expression of p21 in hPSC-derived airway organoids treated with control or 10 mg/ml THBS1 blocking antibody. The yellow arrows highlight the expression of p21 in FOXJ1+ ciliated cells. Scale bar= 50 μm. **i, j,** Immunostaining (i) and quantification (j) of the relative expression of γH2A.X in hPSC-derived airway organoids treated with control or 10 mg/ml THBS1 blocking antibody. The yellow arrows highlight the expression of γH2A.X in FOXJ1+ ciliated cells. Scale bar= 50 μm. N=3 independent biological replicates. Data was presented as mean ± STDEV. *P* values were calculated by unpaired two-tailed Student's t test. **P* < 0.05, ***P* < 0.01.

Extended Data Table 1. Patient Information.

		Gender	Age	COVID	Post-mortem Interval (PMI) (Hours)	Comorbidities	COVID Diagnosis to transplant	Days.hospitalized	[illegible] between specimen obtained and
Non-COVID	#1	M	19	No	N/A	N/A	N/A		1
	#2	F	63	No	N/A	N/A	N/A		1
	#3	M	17	No	N/A	N/A	N/A		Same day
	#4	M	29	No	N/A	N/A	N/A		1
	#5	F	21	No	N/A	N/A	N/A		1
	#6	M	68	No	N/A	N/A	N/A		1
COVID-E	#1	M	61	Yes	N/A	Prior prostate cancer in remission (managed surgically, no chemo/rads)	4 months		1
	#2	M	56	Yes	N/A	HTN, Anxiety	3 months (2 months, 18 days)		1
	#3	F	63	Yes	N/A	Chronic pain after MVA, spinal nerve stimulator	6 months, 1 day		2
	#4	F	51	Yes	N/A	None	3 months		1
	#5	M	64	Yes	N/A	HLD, HLD, DM	12 months		2
	#6	M	70	Yes	N/A	HLD, Pre-DM, TIA	29 months		1
COVID-A	#1	M	70	Yes	4.5		N/A	0	
	#2	M	73	Yes	4		N/A	8	
	#3	F	93	Yes	2.5		N/A	4	
	#4	F	78	Yes	5		N/A	19	
	#5	M	80	Yes	4		N/A	17	
	#6	F	80	Yes	4		N/A	25	
	#7	F	70	Yes	2.5		N/A	43	
	#8	M	72	Yes	4		N/A	48	
	#9	F	69	Yes	3		N/A	41	
	#10	M	58	Yes	6.75		N/A	58	

Extended Data Table 2. Information of antibodies used in immunostaining.

Antibody	Clone #	Host	Catalog #	Vendor	Dilution
Purified anti-human CD68 Antibody	Monoclonal	Mouse	# 333802	Biolegend	1: 100
Anti-alpha smooth muscle Actin antibody	Monoclonal	Mouse	#ab7817	Abcam	1: 400
IL-1B Antibody	Monoclonal	Mouse	#12242S	Cell Signaling	1:500
P21 Antibody	Monoclonal	Rabbit	#2947	Cell Signaling	1: 200
Pro-SP-C Antibody	Polyclonal	Rabbit	# WRAB-9337	Seven Hills Bioreagents	1:500
HT2-280 Antibody	Monoclonal	Mouse	#TB-27AHT2-280	terracebiotech	1:100
FOXJ1 Antibody	Monoclonal	Mouse	#14-9965-82	Invitrogen	1:500
gH2A.X Antibody	Polyclonal	Rabbit	#9718S	Cell Signaling	1:400
Donkey anti-Mouse IgG (H+L) Highly Cross-Adsorbed Secondary Antibody, Alexa Fluor 488	Polyclonal	Donkey	#A-21202	Thermo Fisher Scientific	1:500
Donkey anti-Mouse IgG (H+L) Highly Cross-Adsorbed Secondary Antibody, Alexa Fluor 594	Polyclonal	Donkey	#A-21203	Thermo Fisher Scientific	1:500
Donkey anti-Rabbit IgG (H+L) Secondary Antibody, Alexa Fluor 594 conjugate	Polyclonal	Donkey	#A-21207	Thermo Fisher Scientific	1:500
Donkey anti-Rabbit IgG (H+L) Secondary Antibody, Alexa Fluor 647 conjugate	Polyclonal	Donkey	#A-31573	Thermo Fisher Scientific	1:500
Donkey anti-Mouse IgG (H+L) Secondary Antibody, Alexa Fluor 647	Polyclonal	Donkey	#A-31571	Thermo Fisher Scientific	1:500
Donkey anti-Goat IgG (H+L) Cross-Adsorbed Secondary Antibody, Alexa Fluor 647	Polyclonal	Donkey	#A-21447	Thermo Fisher Scientific	1:500
